# Comparative effectiveness of several adjuvant therapies for patients with hepatocellular carcinoma with high-risk factors for recurrence after hepatectomy: a systematic review and meta-analysis

**DOI:** 10.3389/fmed.2025.1692417

**Published:** 2025-12-30

**Authors:** Zejin Zhao, Haizhao Yi, Yue Xiao, Zhiyan Li, Jinlong Liu

**Affiliations:** 1 Department of Hepatobiliary Surgery, The Affiliated Hospital of Chengde Medical University, Chengde, Hebei, China; 2 Hebei Key Laboratory of Panvascular Diseases, Chengde, Hebei, China

**Keywords:** adjuvant therapy, hepatocellular carcinoma, meta-analysis, recurrence, survival

## Abstract

**Background:**

For patients with hepatocellular carcinoma (HCC) who have high-risk recurrence factors following hepatectomy, there is currently no comprehensive meta-analysis examining the effects of various adjuvant therapies post-resection. The comparative effectiveness of these different adjuvant therapies remains unclear. Consequently, we conducted a review of available trials involving postoperative adjuvant therapies in patients with HCC who underwent radical resection and presented with factors associated with a high risk of recurrence.

**Method:**

We collected all studies on postoperative adjuvant therapies in HCC patients with high-risk recurrence factors, concluding on September 7, 2024, from PubMed, Embase and Web of Science. In these studies, overall survival (OS) and disease-free survival (DFS) were compared between groups by calculating the combined hazard ratios (HRs) and 95% confidence intervals (CIs).

**Result:**

Forty-five eligible trials (8,409 patients) reporting five different therapies were included. Our results indicated that postoperative adjuvant therapies such as TACE, TKI, RT, and HAIC-FOLFOX are effective. In terms of improving the DFS, RT (HR = 0.31, 95%CI: 0.18–0.52) was found to be the most effective adjuvant therapy, followed by TKI (HR = 0.48, 95%CI: 0.36–0.63). Regarding OS improvement, RT (HR = 0.31, 95%CI: 0.19–0.50) demonstrated the highest effectiveness, followed by TKI (HR = 0.50, 95%CI: 0.38–0.66).

**Conclusion:**

Adjuvant therapy following hepatectomy decreases the risk of recurrence, and HCC patients with high risks of recurrence may benefit. TACE, TKI, RT and HAIC-FOLFOX are effective methods for reducing recurrence after HCC with high risks of recurrence. RT appears to be the most effective adjuvant regimen.

## 1 Introduction

Hepatocellular carcinoma (HCC) is one of the most prevalent malignant tumors worldwide, ranking sixth in global incidence and fourth in cancer-related mortality (1). As a leading country in liver cancer cases, China accounts for over half of the world’s annual incidence and mortality associated with liver cancer (2). Curative therapies for HCC include ablation, liver transplantation and radical hepatectomy (3–5). However, ablation is only recommended for patients with early-stage HCC, representing a minority of the overall HCC population. Although liver transplantation offers the best therapy option for HCC patients, the limited availability of donor organs significantly restricts access to this procedure. Hepatic resection remains the primary radical therapy for HCC. Yet, even in early-stage patients, the recurrence rate shortly after surgery remains high (6). The 5-year recurrence and metastasis rate reaches 50–70%, leading to unsatisfactory survival outcomes for most patients following surgery (7, 8). The prognosis is inferior for those with high-risk factors for recurrence (9).

Common high-risk factors for recurrence (10) include vascular invasion (such as vascular thrombus or bile duct thrombus), lymph node metastasis, tumor size, tumor number (≥3), tumor stage, histological grade, satellite metastasis, intact peritoneum status, tumor rupture, alpha-fetoprotein (AFP) levels, degree of cirrhosis, HBV/HCV infection status, and Child-Pugh classification. For HCC patients at high risk of recurrence, the implementation of more aggressive adjuvant therapies postoperatively is essential to reduce recurrence rates. The adjuvant postoperative therapies (11) that are most commonly employed in the present era are as follows: transcatheter arterial chemoembolization (TACE) (12–28), tyrosine kinase inhibitors (TKI) (21, 29–38), hepatic artery infusion chemotherapy (HAIC) (39–43) and radiotherapy (RT) (44–46). Effectively preventing postoperative HCC recurrence has become the key crucial for in patients with HCC (4, 47–49).

Nonetheless, it remains unclear which postoperative adjuvant therapies can significantly improve survival outcomes in HCC patients with high-risk factors for recurrence following hepatectomy alone. Currently, there is lack of meta-analyses summarizing the effectiveness of adjuvant therapy after hepatectomy alone in HCC patients with high-risk recurrence factors. Therefore, the objective of our meta-analysis is to examine this issue in greater detail.

## 2 Materials and methods

### 2.1 Search strategy

A systematic search was conducted in PubMed, Embase, and Web of Science from database inception to September 7, 2024. We followed PRISMA guidelines throughout. The search strategy combined controlled vocabulary and free-text terms, including: (“liver cancer” OR “hepatocellular carcinoma” OR “HCC”) AND (“adjuvant” OR “postoperative” OR “post-resection” OR “after surgery”) AND (“transarterial chemoembolization” OR “TACE” OR “hepatic artery infusion chemotherapy” OR “HAIC” OR “tyrosine kinase inhibitor” OR “TKI” OR “sorafenib” OR “lenvatinib” OR “radiotherapy” OR “RT”). The detailed search strings for each database are provided in Supplementary Table 1 to ensure reproducibility. Reference lists of relevant reviews and included articles were also hand-searched to identify additional eligible studies.

All retrieved records were imported into EndNote software, and duplicates across databases were identified and removed automatically, followed by manual verification. We restricted our search to full-text English-language publications. Conference abstracts, case reports, reviews, and animal studies were excluded. Preprints were considered only if sufficient outcome data were available.

### 2.2 Study selection

Two independent reviewers (Z. Z and H. Y) screened titles and abstracts, followed by full-text review of potentially eligible articles. Discrepancies were resolved by consensus or by consultation with a third reviewer (J. L). Inter-rater agreement was quantified (Cohen’s *κ* = 0.82), indicating strong consistency.

Eligible studies met all of the following criteria: (1) included patients with pathologically or radiologically confirmed HCC undergoing curative-intent hepatectomy. (2) Enrolled populations with at least one high-risk recurrence factor (MVI, PVTT, tumor diameter ≥ 5 cm or multiple tumors). (3) Compared hepatectomy plus postoperative adjuvant therapy (TACE, HAIC, TKI, and RT) with hepatectomy alone. (4) Reported survival outcomes (OS and/or DFS) or sufficient data to estimate hazard ratios (HRs) and 95% confidence intervals (CIs). (5) Study design: randomized controlled trials (RCTs), prospective cohort studies, or retrospective cohort studies.

Exclusion criteria were: (1) duplicate populations (in such cases, the most recent or comprehensive study was retained); (2) single-arm studies without control group; (3) incomplete or non-extractable outcome data; (4) reviews, meta-analyses, case reports, conference abstracts, and animal experiments.

### 2.3 Data extraction and quality assessment

From each eligible study, two reviewers independently extracted data including: study characteristics (author, year, country, design), patient demographics (sample size, age, sex, HBV/HCV status, cirrhosis, AFP levels, tumor features), adjuvant therapy details (type, timing, regimen), and outcomes (OS, DFS). For studies without directly reported HRs, we estimated them using Parmar’s and Tierney’s methods (50, 51).

Quality assessment was performed using the Cochrane risk-of-bias tool for RCTs (52) and the Newcastle–Ottawa Scale (NOS) for cohort studies (53). For NOS, studies scoring ≥7 were considered high quality, 4–6 as moderate, and ≤3 as low quality. Quality assessments were tabulated, and sensitivity analyses restricted to high-quality studies were prespecified.

### 2.4 Statistical analysis

The meta-analysis was conducted using Stata 12.0. Significant heterogeneity was defined as *I*^2^ > 50% or a *Q*-test with *p* < 0.10. A random-effects model was employed when significant heterogeneity was observed; otherwise, a fixed-effects model was selected. A *p*-value < 0.05 indicated a statistically significant result. Pairwise meta-analysis was conducted using R version 4.4.2 (Foundation for Statistical Computing, Vienna, Austria) with R package “meta” (version 8.0–1). Additionally, Bayesian network meta-analysis (NMA) was performed using R (gemtc, rjags) to compare multiple adjuvant modalities simultaneously. Given the clinical and methodological diversity across studies, NMA results were interpreted as exploratory, not definitive.

## 3 Results

### 3.1 Literature search results

**Figure 1:**
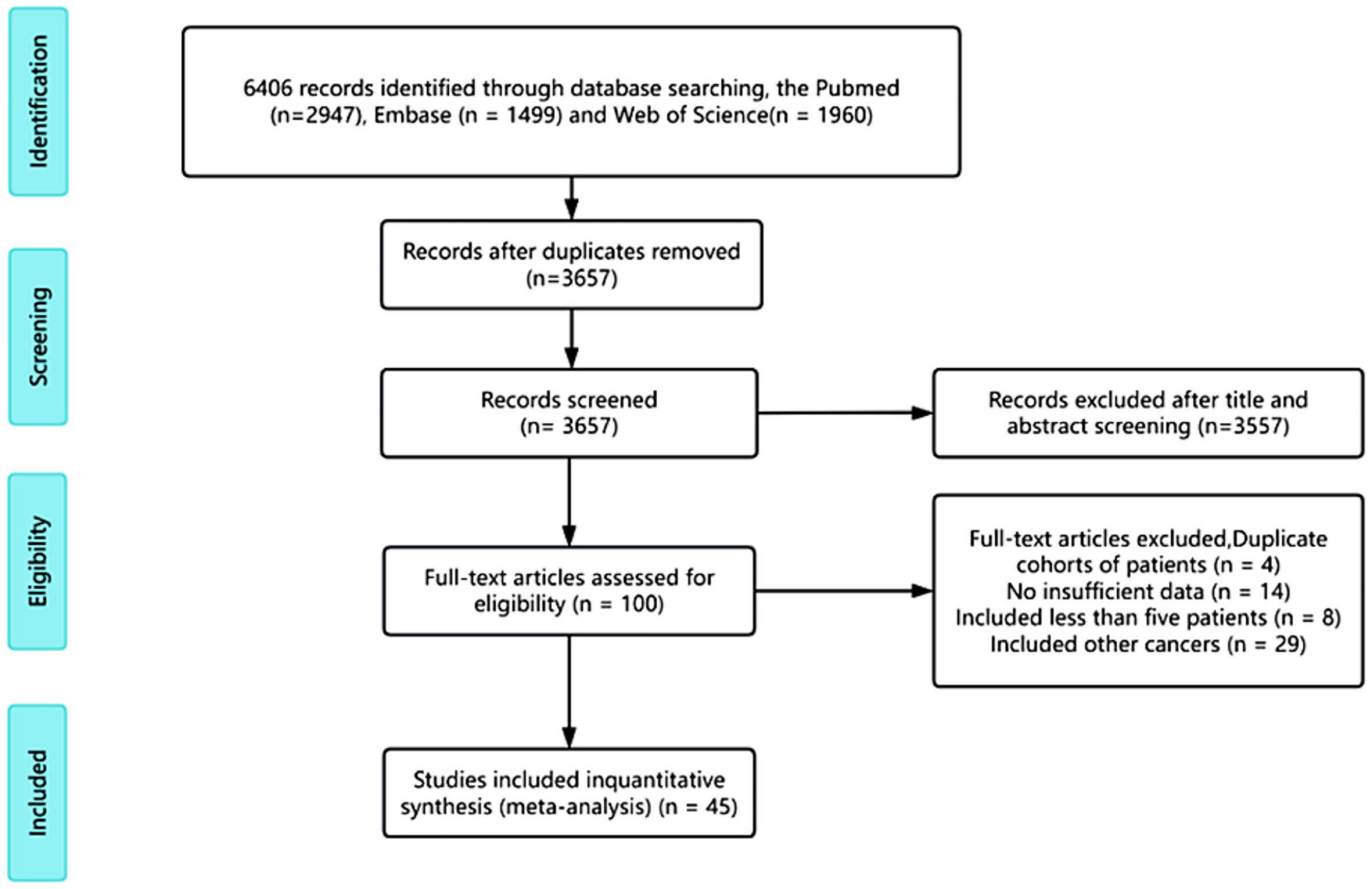
Flowchart outlining the search strategy and details on the studies finally included in the meta-analysis. The diagram summarizes the number of records identified, screened, assessed for eligibility, and ultimately included in the meta-analysis. Reasons for exclusion at each stage—such as duplicate records, insufficient outcome data, non-relevant study design, or overlapping cohorts—are detailed to ensure transparency and reproducibility.

In total, 6,406 articles were identified in PubMed, Embase and Web of Science. After reviewing all titles and abstracts, 6,306 articles were excluded for being irrelevant to the current analysis. The remaining 100 potentially relevant articles underwent a full-text review, resulting in the exclusion of 55 articles for the following reasons: duplicate patient cohorts (*n* = 2), unspecified risk factors (*n* = 33), inclusion of animal experiments (*n* = 8), and inclusion of other cancers (*n* = 12). Finally, 45 articles reporting on 45 trials were included in our meta-analysis. The screening process for selecting studies for inclusion is illustrated in Figure 1.

### 3.2 Quality assessment and study characterization

**Figure 2:**
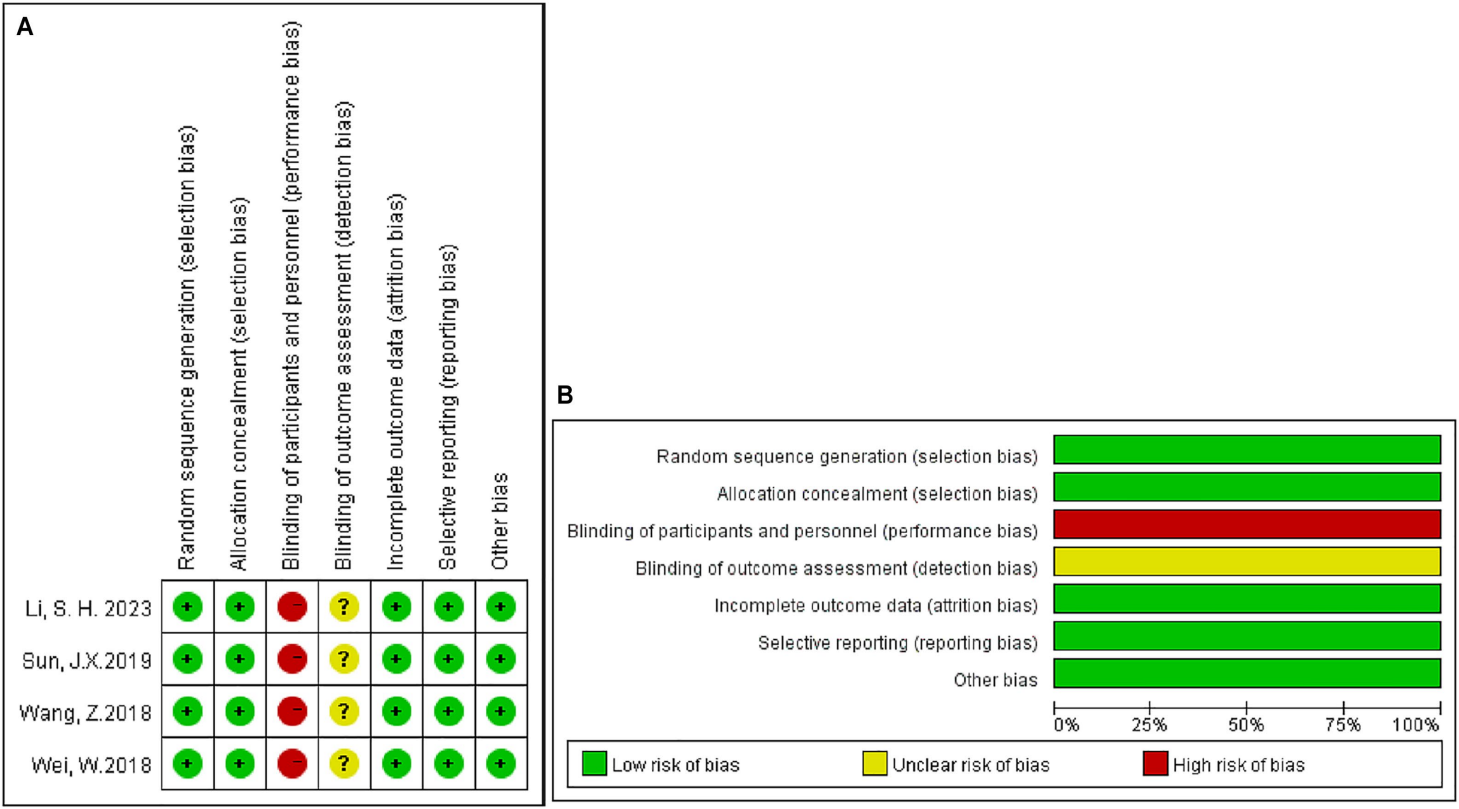
Risk-of-bias assessments for prospective clinical trials included in the meta-analysis. **(A)** Risk-of-bias summary; **(B)** risk-of-bias graph. +: Low risk of bias;?: Unclear risk of bias; −: High risk of bias.

In summary, 45 articles examined 8,409 patients. Of these, 4,166 underwent radical hepatectomy, and 4,243 received adjuvant therapy after the surgery. Adjuvant therapy after surgery consists of TACE, HAIC, RT, and TKI. Pathological or non-invasive methods (imaging, serology, etc.) were used to diagnose HCC in all patients. Liver function was mentioned in all of the trials, and patients with normal liver function or with compensated cirrhosis (Child-Pugh score A or B) were included in the trials. The Eastern Collaborative Oncology Group Physical Performance Status (ECOG PS) of enrolled patients has also been evaluated in some articles (28, 29, 32, 33, 36, 54, 55). We also examined the quality of the studies we included. Among the included studies, 4 were RCTs (28, 43, 44, 56), 39 were retrospective cohort studies (13, 17, 21, 22, 26, 27, 29–33, 39–41, 54, 57–61), and 2 was prospective cohort studies (35, 42). The main characteristics of the studies are summarized in Supplementary Tables 2, 3. All cohort and retrospective studies scored above six on the NOS, indicating medium to high quality (Supplementary Table 4). In terms of the Cochrane Risk of Bias Assessment Tool, all RCTs were deemed to have a low risk of bias (Figure 2). All studies were considered to be of acceptable quality.

### 3.3 Risk factor

Common high-risk factors for recurrence include vascular invasion (either vascular thrombus or bile duct thrombus), lymph node metastasis, maximum tumor diameter ≥ 5 cm, multiple tumors, tumor stage, tumor grade, satellite metastases, the presence of intact peritumor tissue, tumor rupture, invasion of neighboring organs, alpha-fetoprotein (AFP) levels, the degree of cirrhosis, HBV/HCV infection, and Child-Pugh classification.

Relevant high-recurrence factors included in this meta-analysis were MVI (12, 13, 16, 17, 20, 21, 26, 27, 29–31, 33, 35, 40, 42, 43, 45, 55, 61, 62); PVTT (14, 15, 22, 23, 32, 36, 37, 44, 54, 60, 63); maximum tumor diameter ≥ 5 cm (15, 18, 24, 28, 42, 60); multiple tumors (15, 37, 41, 57); and BCLC B-stage HCC (15, 19, 24, 59, 60).

### 3.4 Result of meta-analysis

#### 3.4.1 Postoperative adjunctive HAIC

In total, five studies (39–43) reported prognostic information for hepatectomy alone and postoperative adjunctive application of HAIC in high-risk HCC patients. Risk factors included MVI (39, 40, 42, 43) and multiple tumors (41). Depending on the type of chemotherapeutic agent used, these studies (39–43) can be broadly categorized into two groups: HAIC based on cisplatin (40–42) and HAIC based on FOLFOX (39, 43). All studies (39–43) consisted of postoperative HAIC patients as the experimental group, and all control subjects were patients with hepatectomy alone. Four articles (39, 40, 42, 43) had adjuvant HAIC within 6 weeks postoperatively for all regimens, and one article (41) had adjuvant HAIC within 3 months postoperatively. The chemotherapeutic agents employed were classified as 5-Fu cisplatin (42), IA-call (high-dose cisplatin powder) (40), 5-Fu cisplatin epothilone (41), 5-Fu oxaliplatin (43), and 5-Fu combined with oxaliplatin and mitomycin Cv (39). All studies (39–43) used AFP, and CT/MRI to assess the efficacy after HAIC.

**Figure 3:**
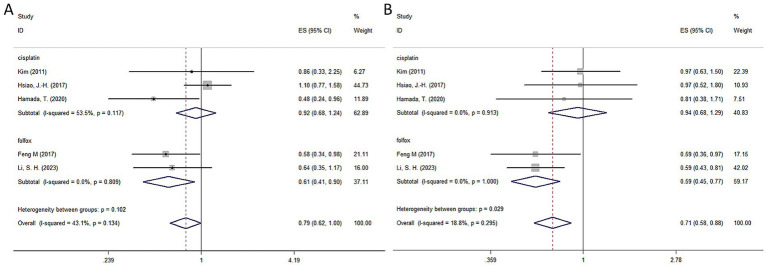
Forest plot of overall survival and disease-free survival for HAIC subgroup of cisplatin-based and folfox-based. **(A)** Overall survival; **(B)** disease-free survival. HAIC, hepatic artery infusion chemotherapy; CI, confidence interval.

The HAIC significantly improved DFS in HCC patients with high-risk factors following hepatectomy, though it did not achieve a statistically significant impact on OS. A fixed-effects model analysis showed that HAIC enhanced DFS (HR = 0.71, 95% CI: 0.58–0.88, *I*^2^ = 18.8%, *p* = 0.295) but had a more limited effect on OS (HR = 0.79, 95% CI: 0.62–1.00, *I*^2^ = 43.1%, *p* = 0.134). Subgroup analysis revealed that the FOLFOX regimen provided substantial survival benefits, significantly improving both DFS (HR = 0.59, 95% CI: 0.45–0.77, *I*^2^ = 0%, *p* = 1.00) and OS (HR = 0.61, 95% CI: 0.41–0.90, *I*^2^ = 0%, *p* = 0.809) with high consistency (*I*^2^ = 0%). In contrast, the 5-Fu cisplatin regimen failed to show significant improvements in DFS (HR = 0.94, 95% CI: 0.68–1.29, *I*^2^ = 0%, *p* = 0.913) or OS (HR = 0.92, 95% CI: 0.68–1.24, *I*^2^ = 53.5%, *p* = 0.117). These results underscore the superiority of the FOLFOX regimen in this context. In conclusion, the FOLFOX regimen is a more effective and consistent postoperative adjuvant HAIC therapy, offering significant improvements in DFS and OS for high-risk HCC patients. It should be prioritized as the preferred regimen in clinical practice. Detailed results are illustrated in Figure 3A (OS) and Figure 3B (DFS).

#### 3.4.2 Postoperative adjunctive RT

Three studies (44–46) on RT were identified that compared individuals with high-risk factors [two with MVI (45, 46) and one with PVTT (44)]. The liver function classification of HCC patients included in the studies were classified as liver function Child-Pugh A/B (44) or liver function Child-Pugh A (45, 46). The year of publication of the included studies ranged from 2008 to 2018. The experimental group of the included studies included patients who received postoperative RT and the control group included in the study consisted of patients who underwent radical hepatectomy. The total amount of radiation was similar in all three studies (44–46) ranging from 50 to 60 Gy, all (44–46) using IMR-T radiotherapy.

**Figure 4:**
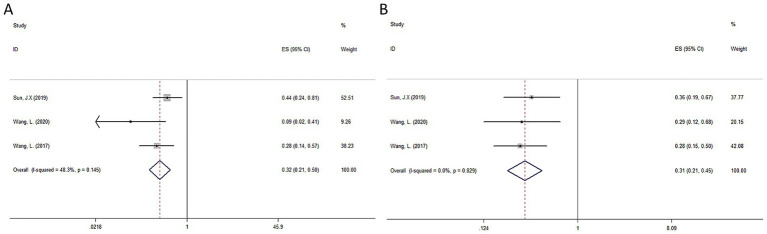
Forest plot of OS **(A)** and DFS **(B)** survival for RT.

All studies (44–46) reported on OS and DFS. The pooled results indicated that postoperative RT significantly improved OS (HR = 0.32, 95% CI: 0.21–0.50, I^2^ = 48.3%, *p =* 0.145) and DFS (HR = 0.31, 95% CI: 0.21–0.45, *I*^2^ = 0%, *p =* 0.829) in HCC with a high risk of recurrence compared to radical hepatectomy alone. The detailed forest plots illustrating the results are presented in Figures 4A,B for OS and DFS.

#### 3.4.3 Postoperative adjunctive TKI

Eight studies (29–33, 36–38) on TKI compared the effects on OS and eight studies (21, 30, 31, 35–38, 54) compared the effects on DFS of TKI and hepatectomy alone in patients with HCC after radical resection with high-risk factors. Risk factors included MVI (21, 29–31, 38, 59), PVTT (32, 36, 54), or both (33, 37) MVI and PVTT. All studies (21, 29–33, 35–38, 54) consisted of HCC patients with postoperative TKI therapy as the experimental group, and all control subjects were patients with hepatectomy alone. Sorafenib was used in all but two studies (29, 30) in which Lenvatinib was used. All 11 studies (21, 29–33, 35–38, 54) administered TKI within 1 month postoperatively, with drug dosages being categorized as Lenvatinib 12 mg/d (≥60 kg) 8 mg/d (<60 kg) (29, 30); Sorafenib 400 mg d12h (31, 33, 35–38, 54); and Sorafenib 200-800 mg/d (32). All liver functions in the included patient population were ≤ Child B.

**Figure 5:**
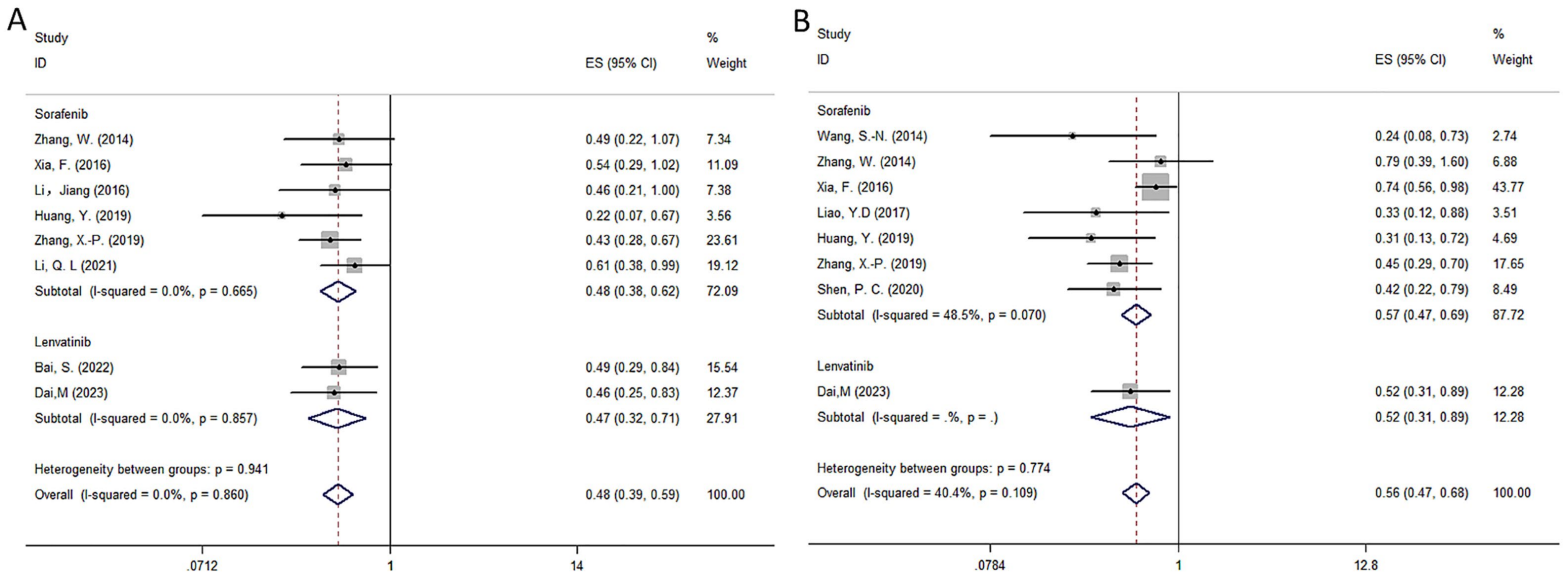
Forest plot of OS **(A)** and DFS **(B)** survival for TKI.

The pooled results indicated that postoperative RT significantly improved OS (HR = 0.48, 95% CI: 0.39–0.59, *I*^2^ = 0%, *p =* 0.860) and DFS (HR = 0.56, 95% CI: 0.47–0.68, *I*^2^ = 40.4%, *p =* 0.109) in HCC with a high risk of recurrence compared to radical hepatectomy alone. The detailed forest plots illustrating the results are presented in Figures 5A,B for OS and DFS.

#### 3.4.4 Postoperative adjunctive TACE

Twenty-eight studies (12–14, 16–28, 46, 55, 57–59, 61, 63, 64) compared the prognosis of adjuvant TACE after hepatectomy alone and hepatectomy alone in HCC patients with high-risk factors for recurrence, of which, 24 studies (12–14, 16–20, 22–24, 26, 27, 46, 55, 57–59, 61, 63) reported OS and 13 studies (12, 13, 16, 17, 20, 21, 25, 26, 28, 46, 55, 61, 64) reported DFS. Risk factors included MVI (12, 13, 16, 17, 20, 21, 25–28, 46, 55, 61, 64), PVTT (14, 22, 23, 63), BCLC B (15, 19, 24, 58, 59), maximum tumor diameter ≥ 5 cm (18) and multiple tumors (56, 57). All studies chose HCC patients with postoperative TACE therapy as the experimental group, and all control subjects were patients with hepatectomy alone.

**Figure 6:**
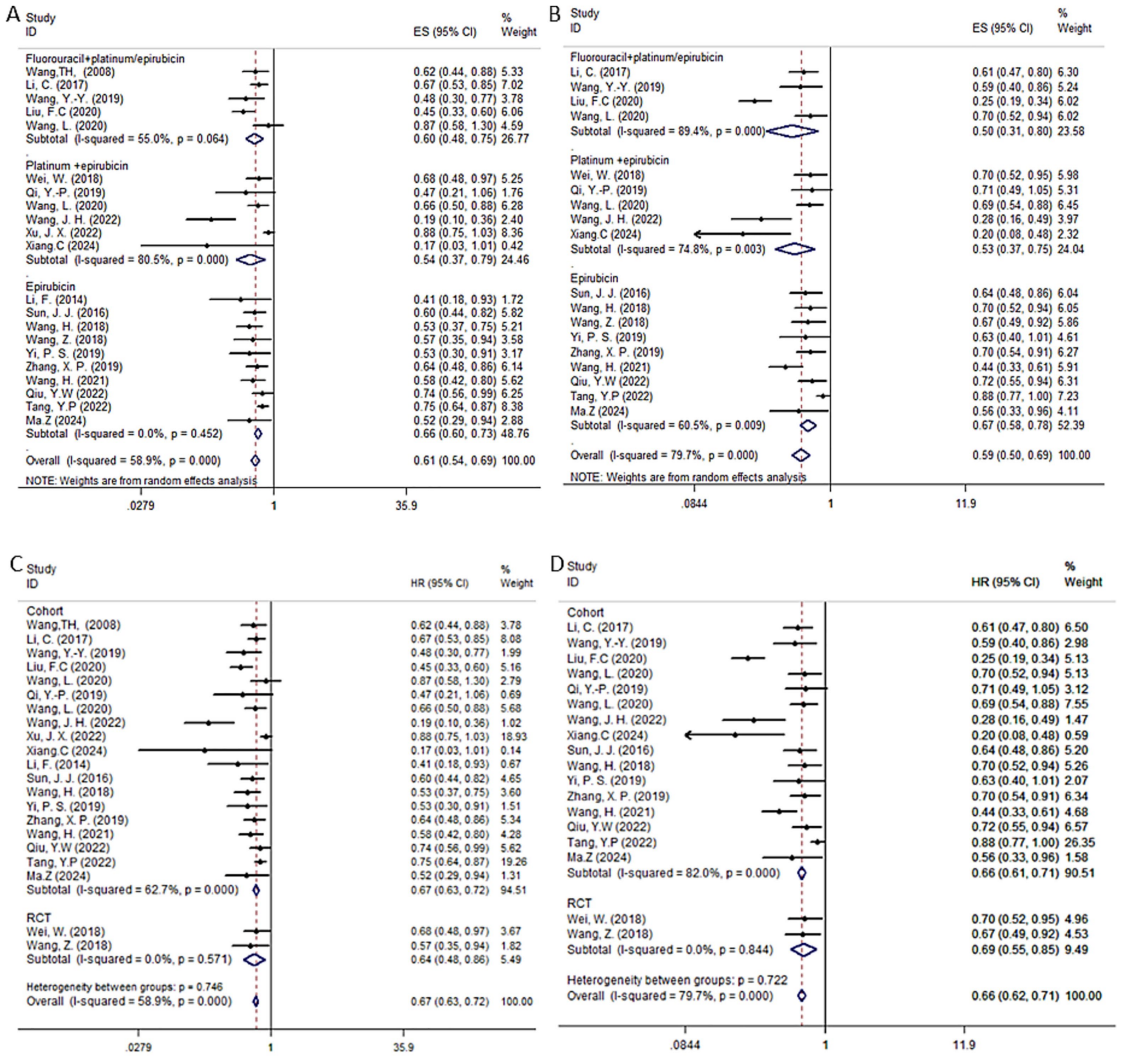
Forest plots of OS **(A,C)** and DFS **(B,D)** in patients receiving postoperative TACE. Subgroup analyses were stratified by study type to assess the consistency of TACE efficacy across different categories of included studies.

The random effects model was employed by the HR of the OS and DFS of postoperative adjuvant TACE. Our results suggested that in HCC patients with high-risk factors, TACE significantly improved OS (HR = 0.61, 95% CI: 0.54–0.69, *I*^2^ = 58.9%, *p* < 0.001) and DFS (HR = 0.59, 95% CI: 0.50–0.69, *I*^2^ = 79.7%, *p <* 0.001) compared to hepatectomy alone. The detailed forest plots illustrating the results are presented in Figures 6A,B for OS and DFS.

We further analyzed the impact of each subgroup on the results. Depending on the TACE strategy used in each study, which was iodinated oil, and the chemotherapeutic agent used, the studies were broadly divided into three categories: fluorouracil in combination with platinum or epirubicin (5 studies) (19, 22, 25, 58, 63); platinum in combination with epirubicin (6 studies) (12, 14, 15, 20, 26, 28); epirubicin alone (10 studies) (13, 16, 18, 23, 24, 26, 55, 56, 59, 61). For OS, the combined HR for fluorouracil plus platinum or epirubicin was 0.6 (95% CI: 0.48–0.75, *I*^2^ = 55.0%, *p =* 0.064); platinum plus epirubicin was 0.57 (95% CI: 0.39–0.83, *I*^2^ = 80.5%, *p <* 0.001); and epirubicin alone was 0.66 (95% CI: 0.60–0.73, *I*^2^ = 0%, *p =* 0.414). All three TACE strategies significantly improved patient OS compared to hepatectomy alone. For DFS, fluorouracil combined with platinum or epirubicin had a combined HR of 0.5 (95% CI: 0.31–0.80, *I*^2^ = 89.4%, *p <* 0.001); platinum combined with epirubicin had a combined HR of 0.6 (95% CI: 0.44–0.82, *I*^2^ = 74.8%, *p =* 0.026); and epirubicin alone had a combined HR of 0.68 (95% CI: 0.58–0.80, *I*^2^ = 60.5%, *p =* 0.007) were all significantly different compared to HR alone, improving patients’ DFS. For the TACE group, we performed a subgroup analysis of RCTs and cohort studies. The results showed that the forest plot trends of the two types of studies were consistent, indicating that the sensitivity analysis results were robust and there was no obvious bias.

#### 3.4.5 Network meta-analysis

**Figure 7:**
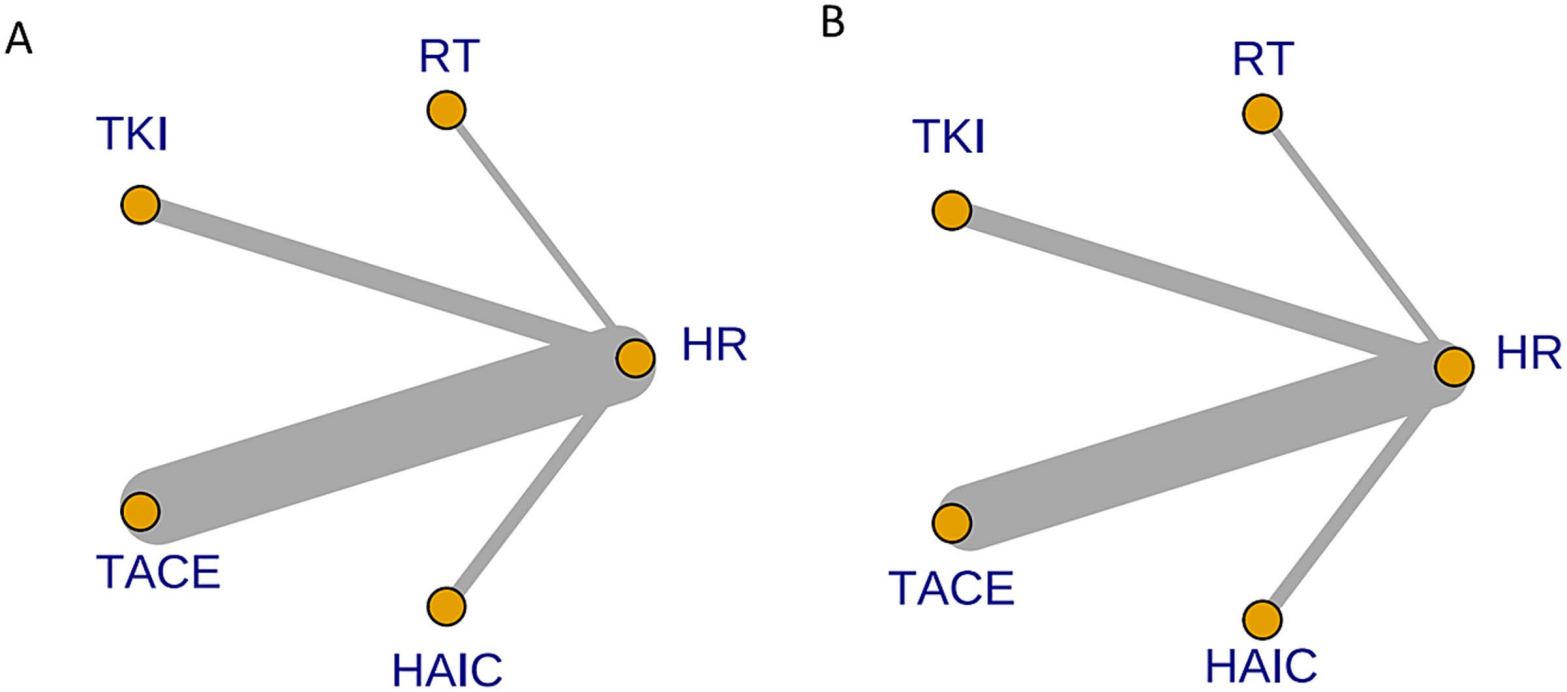
Network diagram of eligible comparisons for OS **(A)** and DFS **(B)**. Each node represents a treatment strategy. Connecting lines indicate direct head-to-head comparisons; line thickness corresponds to the number of studies contributing to each comparison.

Figure 7 depict the comparison networks for OS and DFS, respectively. The width of the edges indicates the number of studies comparing the two treatments, while the size of the nodes represents the number of arms corresponding to each treatment method in the included studies.

**Figure 8:**
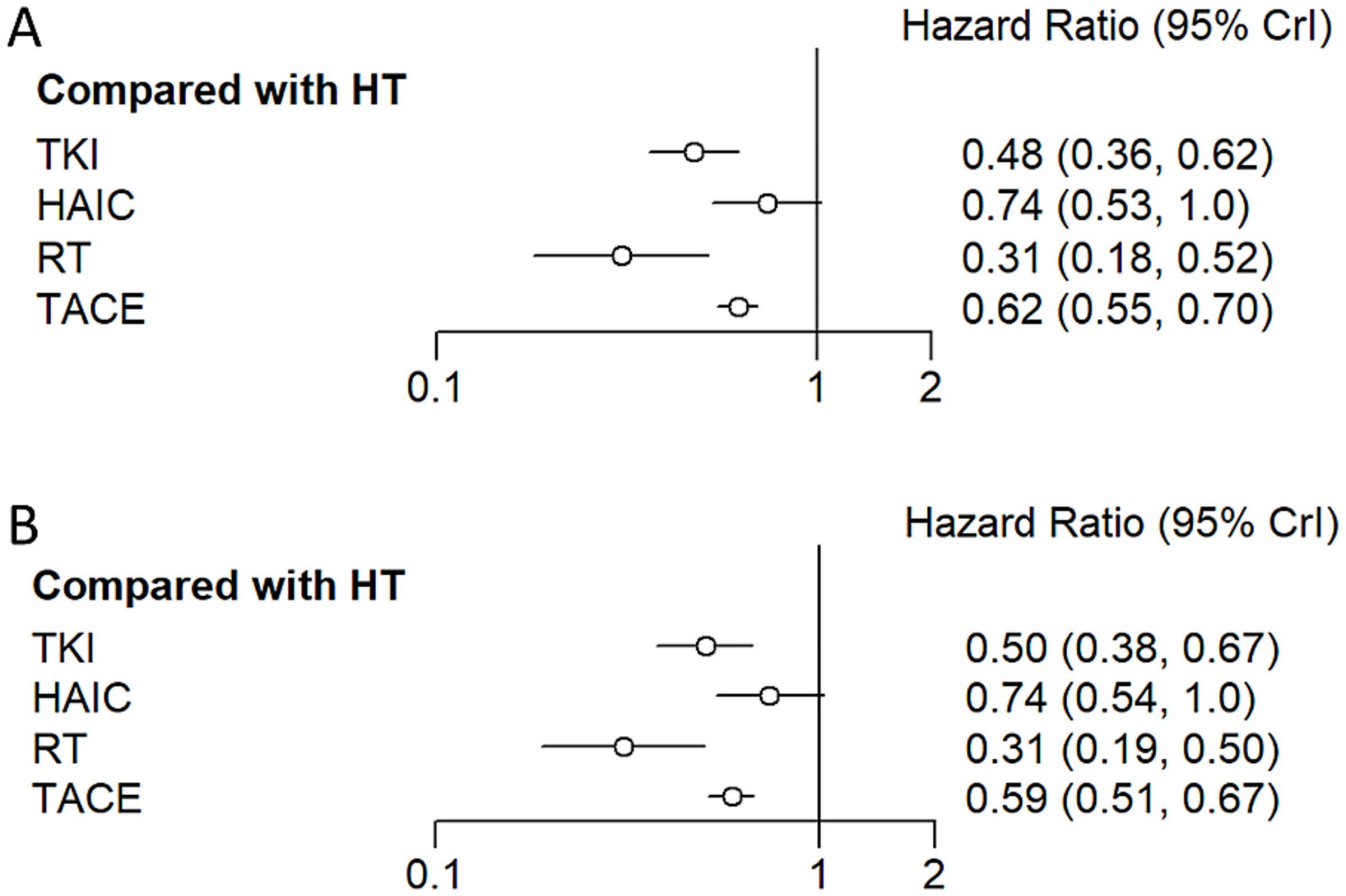
Hazard ratio along with 95% confidence interval for OS **(A)** and DFS **(B)** for each adjuvant therapy compared with hepatectomy.

**Figure 9:**
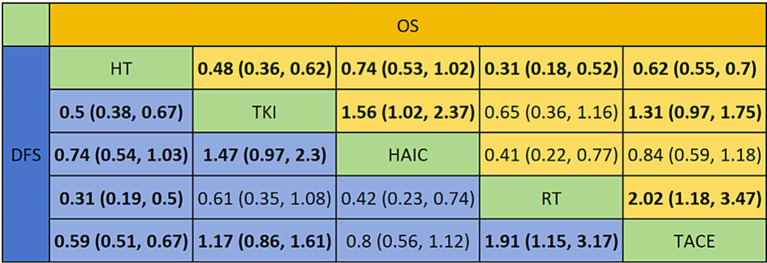
Pooled estimates of the network meta-analysis. This figure presents the synthesized effect estimates for all treatment comparisons within the network meta-analysis, integrating both direct and indirect evidence.

Regarding reducing the OS (Figures 8A, 9), RT (HR = 0.31, 95%CI: 0.18–0.52), TKI (HR = 0.48, 95%CI: 0.36–0.63), TACE (HR = 0.62, 95%CI: 0.55–0.70), and HAIC (HR = 0.74, 95%CI: 0.53–1.0) were all significantly more effective than hepatectomy alone. Furthermore, RT was superior to TACE (HR = 2.02, 95%CI: 1.18–3.47), and TKI was superior to TACE (HR = 1.31, 95%CI: 0.97–1.75) or HAIC (HR = 1.56, 95%CI: 1.02–2.37). The ranking results are presented with RT having the highest likelihood of ranking first for OS, followed by TKI, TACE, and HAIC.

For improving DFS (Figures 8B, 9), patients who underwent RT (HR = 0.31, 95%CI: 0.19–0.50), TKI (HR = 0.50, 95%CI: 0.38–0.66), TACE (HR = 0.59, 95%CI: 0.51–0.67), or HAIC (HR = 0.74, 95%CI: 0.54–1.0) experienced a significantly greater survival benefit compared to those who underwent hepatectomy alone. Notably, RT demonstrated superior efficacy compared to TACE (HR = 1.91, 95%CI: 1.15–3.17), and TKI was superior to TACE (HR = 1.17, 95%CI: 0.86–1.61) or HAIC (HR = 1.47, 95%CI: 0.97–2.3). Among these interventions, RT ranked the highest in terms of improving DFS, followed by TKI, TACE, and HAIC.

### 3.5 Publication bias analysis

We used funnel plots to assess publication bias in the meta-analyses, which revealed that the plots were mostly symmetrical and showed no significant heterogeneity (Supplementary Figure S1).

## 4 Discussion

In recent years, postoperative adjuvant therapy modalities for patients with HCC have evolved rapidly and patient prognosis has improved significantly over time (65). However, current guidelines provide conflicting recommendations regarding adjuvant therapy for HCC following hepatectomy (66–69). Several recent meta-analyses (70–72) have shown that patients with high-risk factors for HCC may benefit from postoperative adjuvant therapy, which should be highly valued and aggressively intervened clinically. It is hoped that the application of postoperative adjuvant therapies such as TACE, HAIC, RT and TKI can stop or delay their recurrence.

This is the first meta-analysis to date, with the largest number of articles included, aimed at evaluating the effectiveness of postoperative adjuvant therapy in patients with HCC with different high-risk factors who have undergone hepatectomy. And provides objective recommendations for the selection of appropriate clinical therapies. This may be important in addressing postoperative adjuvant therapy differences in the selection of HCC patients with high-risk factors for recurrence. This meta-analysis incorporates multiple postoperative adjuvant therapy options and multiple high-risk recurrence factors. Similar meta analyses to our meta-analysis, Chen et al. (70) included only the TACE monoadjuvant population, Huang et al. (71) included only the sorafenib monoadjuvant population, and Pei et al. (72) included only comparisons between different adjuvant therapies in a population with high risk factors for MVI.

In patients with HCC at high risk of recurrence after radical liver cancer resection, although several studies have shown the therapeutic advantages of HAIC for these patients (73, 74), some studies have reached different conclusions. Our results indicate that HAIC seems to improve DFS in HCC patients at high risk of recurrence post-surgery. While, OS did not show significant improvement. Additionally, there are significant differences in the efficacy of different HAIC regimens on OS and DFS. Based on the different chemotherapy agents, these regimens are mainly divided into cisplatin- and oxaliplatin-based treatment plans. This study shows that adjuvant therapy based on the FOLFOX regimen can significantly improve OS and DFS in HAIC patients, while cisplatin-based regimens did not show a clear survival advantage. Cisplatin, as a classic platinum-based drug, has been widely used in the treatment of liver cancer (75). However, with prolonged use, liver cancer cells develop resistance to cisplatin, thereby reducing its efficacy (76). Moreover, cisplatin has notable toxicity, leading to a high incidence of adverse reactions (77). In contrast, oxaliplatin, as a new generation of platinum-based chemotherapy drug, has relatively lower toxicity and can still be effective in cases of cisplatin or carboplatin treatment failure, mainly due to its lower cross-resistance (78). The side effects of the FOLFOX regimen are fewer, especially when used in combination, allowing patients to tolerate longer and more frequent treatments, which may improve overall efficacy. For HCC patients at high risk of recurrence, FOLFOX regimen is safer and more effective than cisplatin (79, 80). In this study, we conducted a comprehensive evaluation of the efficacy of oxaliplatin with a large sample size, consistent with earlier studies with smaller sample sizes, further validating its stable efficacy and safety.

Our results clearly show that compared with surgical resection alone, postoperative application of TKI can significantly improve the OS and DFS of patients, providing a new direction for adjuvant treatment of high-risk HCC patients. It is worth noting that although previous studies have confirmed that sorafenib can reduce the risk of postoperative recurrence of HCC and prolong survival to a certain extent (81), the improvement of DFS in patients with PVTT is not significant (82). This difference may be closely related to the pharmacological characteristics of sorafenib. Sorafenib delays tumor progression mainly by inhibiting the RAF/ MEK/ ERK cascade pathway and weakly blocking VEGFR and PDGFR. However, its ability to remove existing tumor cells is limited, so it is difficult to significantly prolong disease-free survival in groups such as PVTT with high invasiveness and obvious angiogenesis dependence (83). In contrast, lenvatinib has a broader and more potent target inhibition spectrum, including high affinity for VEGFR1-3, FGFR1-4, PDGFRα, RET and KIT. In particular, lenvatinib can simultaneously block VEGF and FGF signaling pathways, which not only strongly inhibits tumor angiogenesis, but also overcomes the FGF-mediated anti-VEGF resistance mechanism (84). This dual-pathway inhibition mode has potential advantages for PVTT patients with highly active angiogenesis and more malignant tumor biological behavior, which may explain their more prominent tumor control ability and better survival outcome in such populations. In general, the differences in target mechanisms provide an important biological basis for the differences in the efficacy of postoperative TKI treatment in PVTT patients.

In China, RT, especially external RT, has been widely used in the treatment of liver cancer. In recent years, the treatment after liver cancer resection has increasingly attracted attention to RT. With advancements in technology, modern radiotherapy techniques such as stereotactic body radiotherapy (SBRT) and intensity-modulated radiotherapy (IMRT) can more precisely target tumor areas while avoiding damage to surrounding healthy tissues (85). The application of these techniques has improved the safety and effectiveness of treatment, allowing high-risk patients to receive higher doses of RT post-surgery without significantly increasing the risk of side effects. This has made RT an important option for preventing tumor recurrence, especially showing great potential in adjuvant therapy after surgery (86). In our meta-analysis, liver cancer patients with high-risk factors, particularly those with MVI and PVTT, showed significant improvement in OS and DFS after receiving RT following radical liver resection. For these high-risk patients, although surgery can remove visible tumor lesions, there remains a high risk of recurrence and local residual disease post-surgery. RT effectively reduces the risk of local recurrence by accurately targeting residual lesions with high precision, thereby controlling the disease and prolonging patient survival. Although the NMA suggests radiotherapy may be the most effective option, this conclusion is drawn from a limited evidence base of only three small studies. Therefore, the findings should be interpreted with utmost caution and cannot be considered definitive.

TACE in patients with HCC exhibiting high-risk recurrence factors such as MVI, PVTT, and large tumors shows significant clinical relevance. Our meta-analysis lies in the comprehensive analysis of various TACE strategies, allowing for a detailed understanding of how to optimize different chemotherapeutic agents to effectively target tumor vascularization. By employing a combination of fluorouracil with platinum-based drugs or epirubicin and assessing their impact on tumor control, we provide evidence that TACE not only obstructs the blood supply to tumors but also delivers locally high concentrations of chemotherapeutic agents, thereby enhancing the cytotoxic effect on residual tumor cells (87, 88). This targeted approach is particularly important for patients with larger tumors, as complete surgical resection is often unachievable, and the risk of postoperative recurrence is heightened (89, 90). Furthermore, TACE’s ability to address multiple tumor lesions through selective arterial embolization underscores its potential as a key therapeutic strategy in managing advanced HCC, ultimately contributing to improved patient prognosis in challenging clinical settings (91). Moreover, the long-term impact of TACE on tumor recurrence and patient quality of life remains underexplored, necessitating further prospective studies to validate our findings and establish comprehensive treatment guidelines for HCC patients with high-risk recurrence factors.

The natural history of HCC exhibits considerable regional heterogeneity, largely attributable to variations in the underlying etiology of liver disease. In Asia, particularly China, HBV infection remains the predominant risk factor for HCC, whereas in Western countries, HCV infection, alcohol-related liver disease, and the rising incidence of NASH represent the leading causes. These etiological disparities influence not only recurrence risk and patterns but also the therapeutic response and applicability of adjuvant strategies. Specifically, HBV-related HCC is associated with a higher propensity for early recurrence, often driven by aggressive tumor biology and mechanisms of intrahepatic metastasis (92). Antiviral therapy has been demonstrated to mitigate this risk, underscoring the critical importance of suppressing viral replication. In contrast, recurrence in metabolic-related HCC, particularly NASH-associated cases, frequently arises from multicentric carcinogenesis, propelled by systemic metabolic dysregulation, chronic inflammation, and the “field effect” of hepatic fibrosis (93–95). Most of the included studies were Asian population. Therefore, interpretation of the present findings must account for geographical and etiological variations, and further validation in non-Asian populations is warranted.

Beyond the postoperative adjuvant therapies investigated in this meta-analysis, biomarkers such as AFP and DCP play a crucial role in disease monitoring and prognostic assessment, providing valuable guidance for tailoring treatment strategies. Furthermore, a comprehensive evaluation of treatment efficacy should incorporate key endpoints like time to recurrence, post-recurrence survival, and quality of life. Future research should prioritize the systematic integration of these metrics to strengthen the evidence base and advance personalized management in hepatocellular carcinoma.

Accumulating evidence indicates that adjuvant therapies, including TKI, radiotherapy, and TACE, can modulate the tumor immune microenvironment in HCC (96–98). TKI, for instance, regulate cytokine secretion, suppress immunosuppressive cell populations such as regulatory T cells and myeloid-derived suppressor cells, and alter the expression of immune checkpoint molecules, thereby potentiating antitumor immunity (99, 100). Radiotherapy can induce immunogenic cell death, enhance tumor antigen presentation, and promote the infiltration of cytotoxic T lymphocytes, fostering a more favorable immune milieu (101, 102). These immunomodulatory effects are of particular relevance in the current era of liver cancer immunotherapy. Combining conventional adjuvant modalities with immune checkpoint inhibitors may synergistically enhance antitumor immune responses, potentially prolonging recurrence-free survival and overall survival. Several ongoing clinical trials are exploring such combination strategies, and a deeper understanding of the underlying mechanisms is essential for optimizing treatment sequencing, dosing, and patient selection.

Several important limitations of the current evidence base should be noted. First, the majority of included studies are retrospective in design, with a limited number of prospective randomized controlled trials available. Second, significant heterogeneity exists in treatment regimens—including variations in chemotherapeutic agents, radiation protocols, and dosing schedules—which may compromise the comparability of outcomes across studies. Third, data are predominantly sourced from specific regions such as East Asia, limiting the generalizability of findings to other populations. Finally, most studies did not incorporate biomarker-based stratification using AFP or DCP, which could help identify patients most likely to derive benefit from adjuvant therapy. These limitations underscore the need for well-designed, prospective, multicenter studies that integrate biomarker-guided stratification.

Additionally, the findings derived from NMA should be interpreted with particular caution. Although NMA is a powerful methodology for comparing multiple interventions, it relies heavily on the underlying assumption of transitivity across the included studies. Given the substantial clinical and methodological heterogeneity in patient populations and treatment protocols, this assumption is often untenable. Presenting treatment rankings as definitive conclusions can be misleading, as indirect comparisons carry an inherent risk of bias. Therefore, the results of NMA in this context should be regarded as exploratory, and their inherent limitations must be explicitly acknowledged.

Notwithstanding these constraints, the present analysis offers valuable insights for clinical practice. For patients with HBV-related HCC in high-incidence regions, our findings support the active consideration of adjuvant therapy, with radiotherapy and TKI emerging as the most promising options based on available evidence. Treatment decisions should be individualized, taking into account liver function, performance status, treatment-related toxicity, and local resource availability and expertise. Although current international guidelines, such as EASL, AASLD, and NCCN, maintain a cautious or reserved stance regarding postoperative adjuvant therapy (66–69), the positive signals observed for TKI and HAIC-FOLFOX in this study provide a rationale for more routine use of adjuvant treatment in high-risk patients and may inform future guideline updates.

Moving forward, efforts should focus on conducting large, multicenter, multi-regional randomized controlled trials to validate these findings, particularly in the field of radiotherapy. Furthermore, as the therapeutic landscape evolves, combination strategies integrating established adjuvant therapies with immune checkpoint inhibitors represent a promising avenue, given their potential for synergistic activation of antitumor immunity. Although such regimens remain beyond the scope of our meta-analysis, emerging evidence suggests that combining targeted therapy with immunotherapy holds considerable promise and warrants future investigation (103). Such investigations will ultimately contribute to refined individualized treatment strategies and improved long-term outcomes for patients with high-risk HCC.

## 5 Conclusion

In summary, adjuvant therapy after hepatectomy can effectively reduce recurrence risk and improve survival outcomes in hepatocellular carcinoma patients with high-risk features. Among the available modalities, including TACE, RT, TKI, and HAIC-FOLFOX, our findings suggest that RT and TKI show particularly favorable benefits as postoperative adjuvant strategies for patients at high risk of recurrence. These modalities may therefore be considered as preferred options in clinical decision-making. Nevertheless, given the retrospective nature and inherent limitations of current evidence, additional high-quality, prospective randomized controlled trials are warranted to further validate the therapeutic value of RT and TKI in this population.

## Data Availability

The original contributions presented in the study are included in the article/Supplementary material, further inquiries can be directed to the corresponding author.
