# Supplementary material for: Comparative effectiveness of several adjuvant therapies for patients with hepatocellular carcinoma with high-risk factors for recurrence after hepatectomy: a systematic review and meta-analysis
Source: Front Med (Lausanne). 2025 Dec 30;12:1692417. doi: 10.3389/fmed.2025.1692417 (PMC12797090; doi:10.3389/fmed.2025.1692417)
Supplement: Supplementary file 1 [file Table_1.docx]

Supplementary Table 1 Search strategy

| **Database** | **Literature search criteria** | **Number of literatures** |
| --- | --- | --- |
| PubMed | ((((Targeted Molecular Therapy) OR (Molecular Therapy, Targeted) OR (Targeted Molecular Therapies) OR (Therapy, Targeted Molecular) OR (Molecular Targeted Therapies) OR (Targeted Therapy, Molecular) OR (Therapy, Molecular Targeted) OR ["Molecular Targeted Therapy" (Mesh) OR (Transcatheter Arterial Infusion) OR (Hepatic Artery Infusion Chemotherapy) OR (chemotherapy) OR (HAIC) OR (transarterial chemoembolization) OR (transcatheter arterial chemoembolization) OR (chemoembolization)OR (TACE) OR（TAI）OR (Radiotherapy) OR (Radiotherapies) OR (Radiation*) OR (RT) OR (Intensity Modulated Radiation Therapy) OR (three-dimensional conformal RT) OR (intensity-modulated radiation therapy) OR (stereotactic body RT) OR (Internal radiation therapy)) AND ((adjuvant) OR (postoperative) OR (prophylactic))) AND (((Hepatectomies) OR (liver resection) OR (hepatic resection) OR (resection) OR ("Hepatectomy" (Mesh))) OR (surgical))) AND ((Carcinomas, Hepatocellular) OR (Hepatocellular Carcinomas) OR (Liver Cell Carcinoma, Adult) OR (Liver Cell Carcinoma) OR (Carcinoma, Liver Cell) OR (Carcinomas, Liver Cell) OR (Cell Carcinoma, Liver) OR (Cell Carcinomas, Liver) OR (Liver Cell Carcinomas) OR (Hepatocellular Carcinoma) OR (Hepatoma) OR (Hepatomas) OR ("Carcinoma, Hepatocellular" (Mesh))) | 2947 |
| Embase | ('carcinoma, hepatocellular':ab,kw,ti OR 'carcinomas, hepatocellular':ab,kw,ti OR 'hepatocellular carcinomas':ab,kw,ti OR 'liver cell carcinoma, adult':ab,ti,kw OR 'adult liver cancer':ab,ti,kw OR 'adult liver cancers':ab,ti,kw OR 'liver cell carcinoma':ab,ti,kw OR 'carcinoma, liver cell':ab,ti,kw OR 'carcinomas, liver cell':ab,ti,kw OR 'cell carcinoma, liver':ab,ti,kw OR 'cell carcinomas, liver':ab,ti,kw OR 'liver cell carcinomas':ab,ti,kw OR 'hepatocellular carcinoma':ab,ti,kw OR 'hepatoma':ab,ti,kw OR 'hepatomas':ab,ti,kw OR 'liver cell carcinoma'/exp) AND ('hepatectomies':ab,kw,ti OR 'hepatectomy':ab,kw,ti OR 'hepatectomy'/exp OR 'resection':ab,kw,ti OR 'surgical':ab,kw,ti) AND ('adjuvant'/exp OR 'adjuvans':ab,kw,ti OR 'bacterial adjuvant':ab,kw,ti OR 'oil adjuvant':ab,kw,ti OR 'adjuvant':ab,kw,ti OR 'prophylactic':ab,kw,ti OR 'postoperative':ab,ti) AND ('radiotherapy'/exp OR 'irradiation':ab,kw,ti OR 'radiation':ab,kw,ti OR 'radiotherapy':ab,kw,ti OR 'radiotherapies':ab,kw,ti OR 'rt':ab,kw,ti OR 'radiations':ab,kw,ti OR 'therapy, targeted molecular':ab,ti,kw OR 'targeted molecular therapies':ab,ti,kw OR 'molecular therapy, targeted':ab,ti,kw OR 'targeted molecular therapy':ab,ti,kw OR 'therapy, molecular targeted':ab,ti,kw OR 'molecular':ab,ti,kw OR 'targeted therapy':ab,ti,kw OR 'molecular targeted therapies':ab,ti,kw OR 'molecularly targeted therapy'/exp OR 'molecularly targeted therapy':ab,kw,ti OR 'hepatic artery infusion chemotherapy':ab,ti,kw OR 'transcatheter arterial infusion':ab,ti,kw OR 'haic':ab,ti,kw OR 'chemoembolization'/exp OR 'chemoembolization':ab,kw,ti OR 'transcatheter arterial chemoembolization':ab,ti,kw OR 'transarterial chemoembolization':ab,ti,kw OR 'tace':ab,ti,kw OR 'tai':ab,ti,kw) | 1499 |
| Web of Science | ((ALL=(postoperative OR adjuvant OR adjuvan* OR prophylactic)) AND ALL=(Hepatectomy OR hepatectomies OR hepatectomy OR resection* OR surgical)) AND ALL=(Carcinomas, Hepatocellular OR Hepatocellular Carcinoma* OR Liver Cell Carcinoma, Adult OR Liver Cancer*, Adult OR Adult Liver Cancer* OR Cancer*, Adult Liver OR Liver Cell Carcinoma* OR Carcinoma*, Liver Cell OR Cell Carcinoma*, Liver OR Hepatoma*) AND ((Molecular Targeted Therapies OR Targeted Therapy OR Molecular Therapy OR Molecular Targeted OR Targeted Molecular Therapy OR Molecular Therapy, Targeted OR Targeted Molecular Therapies OR Therapy, Targeted Molecular OR Molecular Targeted Therapy)) OR (((Transcatheter Arterial Infusion) OR (Hepatic Artery Infusion Chemotherapy) OR (chemotherapy) OR (HAIC) OR (transarterial chemoembolization) OR (transcatheter arterial chemoembolization) OR (chemoembolization) OR (TACE) OR (TAI))) OR(((Radiotherapies) OR (Radiation*) OR (RT) OR (Radiotherapies))) | 1960 |

Supplementary Table 2 Basic characteristics and quality assessment of the included studies

| Auther | Year | Country | Center (S/M) | Study period | Study design | Intervention | Main outcome | PSM (Y/N) | NOS score |
| --- | --- | --- | --- | --- | --- | --- | --- | --- | --- |
| [Kim](44\\KIM2011.pdf) | 2011 | Korea | S | 2006.01-2008.12 | Prospective | HAIC-HR | OS DFS | N | H |
| [Hsiao, J.-H.](44\\HSIAO2017.pdf) | 2017 | China | S | 2006-2014 | Retrospective | HAIC-HR | OS DFS | N | H |
| [Hamada, T.](44\\HAMADA2020.pdf) | 2020 | Japan | S | 2004–2014 | Retrospective | HAIC-HR | OS DFS | N | H |
| [Feng,M](44\\LI S2020.pdf) | 2017 | China | S | 2005.5-2010.5 | Retrospective | HAIC-HR | OS DFS | Y | H |
| [Li, S. H.](44\\LI SH2023.pdf) | 2023 | China | M | 2016.6-2021.8 | RCT | HAIC-HR | OS DFS | N | H |
| [Sun, J.X](44\\SUN JX2019.pdf) | 2019 | China | S | 2013.07-2016.06 | RCT | RT-HR | OS DFS | N | H |
| [Wang, L.](44\\WANG L2020 (2).pdf) | 2020 | China | S | 2015.07-2018.12 | Retrospective | RT-HR | OS DFS | N | H |
| [Wang, S.-N.](44\\WANG SN 2014.pdf) | 2014 | China | S | 2010.5-2012.11.30 | Prospective | Sorafenib+HR | OS DFS | N | H |
| [Zhang, W.](44\\zhang w 2014.pdf) | 2014 | China | S | 2009.8.1-2011.12.31 | Retrospective | Sorafenib+HR | OS DFS | N | H |
| [Xia, F.](44\\XIA F 2016.pdf) | 2016 | China | S | 2010.09–2013.09 | Retrospective | Sorafenib+HR | OS DFS | N | M |
| [Li，Jiang](44\\Li Jiang2016.pdf) | 2016 | China | S | 2009.01–2013.12 | Retrospective | Sorafenib+HR | OS | N | H |
| [Liao, Y.D](44\\LIAO YD2017.pdf) | 2017 | China | S | 2010.07–2013.07 | Retrospective | Sorafenib+HR | DFS | N | H |
| [Huang, Y.](44\\HUANG Y2019.pdf) | 2019 | China | S | 2009.01–2016.12 | Retrospective | Sorafenib+HR | OS DFS | N | H |
| [Zhang, X.-P.](44\\ZHANG XP2019.pdf) | 2019 | China | S | 2009–2016 | Retrospective | Sorafenib+HR | OS DFS | N | H |
| [Li, Q. L](44\\LI QL2021.pdf) | 2021 | China | S | 2009.08–2017.08 | Retrospective | Sorafenib+HR | OS PFS | Y | H |
| [Bai, S.](44\\Bai 2022.pdf) | 2022 | China | S | 2019.6.1-2021.6.1 | Retrospective | Lenvatinib+HR | OS DFS | N | H |
| Dai,M | 2023 | China | S | 2019.1-2022.1 | Retrospective | Lenvatinib+HR | OS DFS | Y | H |
| [Wang,TH,](44\\WANG TH2008.pdf) | 2008 | China | S | 1997.01-2004.12 | Retrospective | TACE+HR | OS | N | H |
| [Li, F.](44\\LI F2015.pdf) | 2014 | China | S | 2006.02-2009.05 | Retrospective | TACE+HR | OS | N | H |
| [Dong, Z. R.](44\\Dong 2015.pdf) | 2015 | China | S | 2004.01-2008.12 | Retrospective | TACE+HR | OS DFS | N | H |
| [Sun, J. J.](44\\SUN JJ2015.pdf) | 2016 | China | S | 2004.01-2013.6 | Retrospective | TACE+HR | OS DFS | N | H |
| [Li, C.](44\\LI C2017.pdf) | 2017 | China | S | 2007-2013 | Retrospective | TACE+HR | OS DFS | Y | M |
| [Ye, J. Z.](44\\Ye2017.pdf) | 2017 | China | S | 2012.01-2015.12 | Retrospective | TACE+HR | OS DFS | N | H |
| [Wang, H.](44\\WANG H2018.pdf) | 2018 | China | S | 2010.01-2014.12 | Retrospective | TACE+HR | OS DFS | N | H |
| [Wang, Z.](44\\WANG Z2018.pdf) | 2018 | China | S | 2011.08-2014 | RCT | TACE+HR | OS DFS | N | H |
| [Wei, W.](44\\WEI W2018.pdf) | 2018 | China | S | 2009.06-2012.12 | RCT | TACE+HR | OS DFS | N | H |
| [Liu, S.](44\\LIU S2019.pdf) | 2019 | China | S | 2007.01-2012.12 | Retrospective | TACE+HR | OS | Y | H |
| [Qi, Y.-P.](44\\Qi2019.pdf) | 2019 | China | S | 2012.01-2014.12 | Retrospective | TACE+HR | OS DFS | N | H |
| [Wang, Y.-Y.](44\\WANG YY2019.pdf) | 2019 | China | S | 2004.09-2015.12 | Retrospective | TACE+HR | OS DFS | Y | H |
| [Yi, P. S.](44\\YI PS2019.pdf) | 2019 | China | S | 2009.01-2012.10 | Retrospective | TACE+HR | OS DFS | N | H |
| [Zhang, X. P.](44\\Zhang 2019.pdf) | 2019 | China | S | 2002.1-2015.12 | Retrospective | TACE+HR | OS DFS | N | H |
| [Liu, F.C](44\\LIU FC2020.pdf) | 2020 | China | S | 2008.01-0213.05 | Retrospective | TACE+HR | OS DFS | N | H |
| [Wang, L.](44\\WANG L2020.pdf) | 2020 | China | S | 2013.10-2015.10 | Retrospective | TACE+HR | OS DFS | Y | H |
| [Wang, L.](44\\WANG L2021.pdf) | 2020 | China | S | 2012.12-2015.12 | Retrospective | TACE+HR | OS DFS | Y | H |
| [Wang, H.](44\\WANG H2021.pdf) | 2021 | China | S | 2009.12-2010.12 | Retrospective | TACE+HR | DFS | Y | H |
| [Qiu, Y.W](44\\QIU2022.pdf) | 2022 | China | S | 2014.04-2019.07 | Retrospective | TACE+HR | OS DFS | Y | H |
| [Tang, Y.P](44\\TANG YP2022.pdf) | 2022 | China | M | 2011.01-2015.12 | Retrospective | TACE+HR | OS DFS | N | M |
| [Wang, J. H.](44\\Wang2022.pdf) | 2022 | China | S | 2007.7-2009.1 | Retrospective | TACE+HR | OS DFS | N | H |
| [Xu, J. X.](44\\Xu2022.pdf) | 2022 | China | S | 2012.1-2017.12 | Retrospective | TACE+HR | OS DFS | N | H |
| Xiang,C | 2024 | China | S | 2013.1-2019.12 | Retrospective | TACE+HR | OS DFS | Y | H |
| Ma.Z | 2024 | China | S | 2015.12-2018.12 | Retrospective | TACE+HR | OS DFS | N | H |
| [Wang, L.](44\\WANG LM2017.pdf) | 2017 | China | S | 2008.7-2015.12 | Retrospective | TACE RT HR | OS DFS | N | H |
| [Wang, L.](44\\Wang2019.pdf) | 2019 | China | S | 2008.7-2016.12 | Retrospective | TACE RT HR | OS DFS | Y | H |
| [Shen, P. C.](44\\SHEN PC2020.pdf) | 2020 | China | S | 2015.8-2018.1 | Retrospective | TACE Sorafenib HR | OS DFS | N | H |
| [Lin, K.](44\\Lin2-022.pdf) | 2022 | China | S | 2014.2-2021.1 | Retrospective | TACE TKI HR | OS DFS | Y | H |

S, single; M, multiple; OS, overall survival time; DFS, disease-free survival time; RCT, randomized controlled trial; TACE, transhepatic arterial chemoembolization; HAIC, hepatic artery infusion chemotherapy; RT, radiotherapy; PA-, Postoperative adjuvant-; M, medium; H, high; PSM, propensity score matching; Y, yes; N, no.

Supplementary Table 3

| Author  (Year) | Treatments | Patients | Age  (years) | Male/Female | HBV  (+/-) | HCV  (+/-) | Cirrhosis  (+/-) | AFP  (ng/ml) | BCLC  (0+A)/B/C | Child  -pugh Class  (A/B) | Tumor number  (S/M) | Tumor  Size  (cm) | MVI  (+/-) |
| --- | --- | --- | --- | --- | --- | --- | --- | --- | --- | --- | --- | --- | --- |
| [Kim 2011](44\\KIM2011.pdf) | HAIC | 31 | 51.1±9.4 | 24/7 | 25/6 | 1/30 | 10/21 | 156.40 (1.20–34,089.00) | (TNM I/II/III/IVa)  2/18/9 /2 | NA | 29/2 | 4.80±2.30 | 25/6 |
|  | HR | 62 | 55.2±8.2 | 50/12 | 50/12 | 2/30 | 36/26 | 77.20  (1.50–30,676.00) | (TNM I/II/III/IVa)  6/41/12 /3 | NA | 60/2 | 4.30±2.40 | 43/19 |
| [Hsiao, J.-H. 2017](44\\HSIAO2017.pdf) | HAIC | 61 | 58.0  (24.0–77.0) | 51/10 | 38/23 | 10/51 | NA | NA | pTNM  pT2 25  pT3a 17  pT3b 14  pT4 5 | 28/33 | NA | ≤5-20  >5-41 | NA |
|  | HR | 160 | 63.3  (27.0–86.0) | 126/34 | 78/83 | 51/109 | NA | NA | pTNM  pT2 97  pT3a 21  pT3b 12  pT4 30 | 89/71 | NA | ≤5-81  >5-79 | NA |
| [Hamada, T. 2020](44\\HAMADA2020.pdf) | HAIC | 37 | 65.0±9.0 | 27/10 | Hepatitis(+/-)19/18 | NA | 14/23 | NA | 0/0/37 | 35/2 | 28/9 | 5.60±3.70 | 37/0 |
|  | HR | 85 | 68.0±9.0 | 68/17 | Hepatitis(+/-)57/28 | NA | 42/43 | NA | 0/0/85 | 73/12 | 54/31 | 5.40±3.60 | 62/23 |
| [Feng,M 2017](44\\LI S2020.pdf) | HAIC | 42 | 59.1±6.2 | 31/11 | 25/17 | 8/34 | 32/10 | 562.40±54.10 | NA | 24/18 | 24/18 | 6.20±1.50 | 28/14 |
|  | HR | 43 | 58.4±5.7 | 30/13 | 24/19 | 7/36 | 33/10 | 547.50±49.20 | NA | 27/16 | 23/20 | 5.70±1.30 | 25/18 |
| [Li, S. H. 2023](44\\LI SH2023.pdf) | HAIC | 157 | 50.0(25.0-75.0) | 136/21 | 137/20 | 11/146 | 79/78 | 164.6  (0.87-121000.00) | NA | 143/14 | 114/43 | 5.60(1.80-30.00) | 157/0 |
|  | HR | 158 | 54.0(27.0-75.0) | 139/19 | 138/20 | 152/6 | 84/79 | 189.1  (1.28-121000.00) | NA | 143/15 | 128/30 | 5.40(1.50-16.00) | 158/0 |
| [Sun, J.X 2019](44\\SUN JX2019.pdf) | RT | 26 | 49.6 ± 7.7 | 24/2 | 24/2 | 0/26 | NA | <400 11  ≥400 15 | 0/0/26 | NA | 25/1 | ≤5 4  >5 22 | NA |
|  | HR | 26 | 51.1 ± 10.8 | 24/2 | 24/2 | 0/26 | NA | <400 7  ≥400 19 | 0/0/26 | NA | 24/2 | ≤5 1  >5 25 | NA |
| [Wang, L. 2020](44\\WANG L2020 (2).pdf) | RT | 29 | 55.9 ± 8.1 | 24/5 | 24/5 | NA | 15/14 | ≤400 22  >400 7 | NA | 29/0 | 27/2 | 4.75 ± 2.15 | 29/0 |
|  | HR | 30 | 56.6 ± 9.4 | 25/5 | 27/3 | NA | 18/12 | ≤400 23  >400 7 | NA | 30/0 | 28/2 | 4.50 ± 2.98 | 30/0 |
| [Wang, S.-N. 2014](44\\WANG SN 2014.pdf) | Sorafenib | 14 | 61.4±10.2 | 13/1 | 10/4 | 13/1 | 11/3 | 2035.55± 3636.82 | NA | NA | 14/0 | 6.26±2.21 | 11/3 |
|  | HR | 17 | 59.7±11.3 | 15/2 | 13/4 | 5/12 | 16/1 | 1755.44± 5636.58 | NA | NA | 17/0 | 5.51±3.30 | 7/10 |
| [Zhang, W. 2014](44\\zhang w 2014.pdf) | Sorafenib | 32 | 51.7±1.4 | 25/7 | 28/ NA/4 | NA | NA | ≤20 16  >20 16 | 0/22/10(TNM) | 32/0 | 21/11 | 5.70±0.60 | 32/0 |
|  | HR | 46 | 54.5±1.6 | 42/4 | 38/8 | NA | NA | ≤20 22  >20 24 | 0/26/20  (TNM) | 46/0 | 28/18 | 7.70±0.80 | 46/0 |
| [Xia, F. 2016](44\\XIA F 2016.pdf) | Sorafenib | 34 | 48(21-78) | 25/9 | 29/5 | NA | 30/4 | ≤400 11  >400 23 | 0/0/34 | 27/7 | 2 (1-8) | 6.40(2.80-20.20) | NA |
|  | HR | 68 | 57(18-79) | 50/18 | 59/9 | NA | 60/8 | ≤400 23  >400 45 | 0/0/68 | 54/14 | 2 (1-10) | 5.9(2.9-21.3) | NA |
| [Li，Jiang 2016](44\\Li Jiang2016.pdf) | Sorafenib | 12 | 49.8±6.5 | 12/0 | 12/0 | NA | 10/2 | <400 4  ≥400 8 | 0/0/12 | NA | NA | 9.80±2.10 | NA |
|  | HR | 24 | 52.8±6.9 | 24/0 | 24/0 | NA | 21/3 | <400 6  ≥400 18 | 0/0/24 | NA | NA | 11.20±2.50 | NA |
| [Liao, Y.D 2017](44\\LIAO YD2017.pdf) | Sorafenib | 14 | 47.4±10.6 | 11/3 | 11/3 | NA | NA | ≤400 7  >400 7 | 1/0/13 | 13/1 | 9/5 | <10 10  ≥10 4 | 14/0 |
|  | HR | 28 | 48.4±11.0 | 26/2 | 27/1 | NA | NA | ≤400 13  >400 15 | 3/1/24 | 25/3 | 23/5 | <10 18  ≥10 10 | 28/0 |
| [Huang, Y. 2019](44\\HUANG Y2019.pdf) | Sorafenib | 16 | 52.3±11.9 | 12/4 | 14/2 | NA | NA | ≤400 8  >400 8 | 14/2/0 | 16/0 | 14/2 | ≤5 6  >5 10 | 16/0 |
|  | HR | 33 | 51.3±11.9 | 30/3 | 26/7 | NA | NA | ≤400 19  >400 14 | 29/4/0 | 31/2 | 30/3 | ≤5 16  >5 17 | 33/0 |
| [Zhang, X.-P. 2019](44\\ZHANG XP2019.pdf) | Sorafenib | 113 | 49.0(43.0-56.0) | 97/16 | 102/9 | 3/110 | 69/44 | 385.00 (30.00-1210.00) | 88/25/0 | 111/2 | 96/17 | 5.90(4.00-9.00) | 113/0 |
|  | HR | 113 | 48.0(40.0-57.0) | 98/15 | 98/15 | 3/110 | 82/31 | 472.30 (26.10-1210.00) | 75/38/0 | 112/1 | 92/21 | 5.42(3.8-9.1) | 113/0 |
| [Li, Q. L 2021](44\\LI QL2021.pdf) | Sorafenib | 42 | 54.2±1.4 | 34/8 | Hepatitis(+/-)  33/9 | NA | 34/8 | ≤20 21  >20 21 | 16/14/7  BCLC 0--5 | 41/0 | 17/25 | 6.20±0.60 | 42/0 |
|  | HR | 42 | 54.6±1.7 | 35/7 | Hepatitis(+/-)  34/8 | NA | 37/5 | ≤20 21  >20 21 | 16/15/9  BCLC 0--2 | 41/0 | NA | 7.20±0.80 | 42/0 |
| [Bai, S. 2022](44\\Bai 2022.pdf) | Lenvatinib | 57 | 52 (21-69) | 52/5 | 37/20 | NA | 27/30 | ≤400 33  >400 24 | NA | NA | 43/14 | 17/40 | 33/24 |
|  | HR | 57 | 53 (20-70) | 46/11 | 38/19 | NA | 20/37 | ≤400 33  >400 24 | NA | NA | 45/12 | 16/41 | 31/26 |
| Dai,M 2023 | Lenvatinib | 31 | ＞60 6 | 31/0 | 25/6 | NA | 17/14 | ≤400 11  >400 20 | NA | 28/3 | 24/7 | >5 18 | NA |
|  | HR | 31 | ＞60 5 | 27/4 | 26/5 | NA | 21/10 | ≤400 10  >400 21 | NA | 28/3 | 21/10 | >5 18 | NA |
| [Wang,TH,  2008](44\\WANG TH2008.pdf) | TACE | 90 | Median 45.0 | 76/14 | 79/11 | NA | 65/14 | ≤400 32  >400 18 | 0/74/0 | 70/4 | 0/74 | 5.68±2.83 | 0/74 |
|  | HR | 79 | Median 50.0 | 67/12 | 67/12 | NA | 61/13 | ≤400 47  >400 27 | 0/61/0 | 56/5 | 0/61 | 5.54±3.13 | 0/61 |
| [Li, F. 2014](44\\LI F2015.pdf) | TACE | 26 | 53.6±10.2 | 22/4 | 26/0 | 0/26 | 17/9 | ≤400 17  >400 9 | 0/26/0 | 12/14 | 22/4 | 4.89±2.81 | NA |
|  | HR | 34 | 51.6±9.4 | 30/4 | 34/0 | 0/34 | 21/13 | ≤400 16  >400 18 | 0/34/0 | 16/18 | 26/8 | 5.07±3.13 | NA |
| [Dong, Z. R. 2015](44\\Dong 2015.pdf) | TACE | 74 | ≤53 41  >53 33 | 67/7 | 68/6 | NA | 61/13 | ≤400 47  >400 27 | 0/74/0 | 70/4 | 0/74 | 5.68±2.83 | 0/74 |
|  | HR | 61 | ≤53 26  >53 35 | 57/4 | 58/3 | NA | 49/12 | ≤400 37  >400 24 | 0/61/0 | 56/5 | 0/61 | 5.54±3.13 | 0/61 |
| [Sun, J. J. 2016](44\\SUN JJ2015.pdf) | TACE | 137 | 49.9±0.7 | 120/17 | 121/16 | NA | 61/76 | ≤400 79  >400 58 | BCLC 0--61  BCLC A+B 76 | 135/2 | 126/11 | 6.51±0.26 | 137/0 |
|  | HR | 185 | 48.9±0.9 | 167/18 | 163/22 | NA | 87/98 | ≤400 94  >400 91 | BCLC 0--87  BCLC A+B 98 | 183/2 | 168/17 | 6.99±0.29 | 185/0 |
| [Li, C. 2017](44\\LI C2017.pdf) | TACE | 284 | 50.6±11.1 | 231/53 | 279/5 | 5/279 | 256/28 | ≤400 139  >400 145 | 176/45/63 | NA | 223/61 | 8.20±3.30 | 126/158 |
|  | HR | 284 | 50.9±12.3 | 236/48 | 279/5 | 5/279 | 255/29 | ≤400 146  >400 138 | 178/45/61 | NA | 221/63 | 8.20±3.30 | 117/167 |
| [Ye, J. Z. 2017](44\\Ye2017.pdf) | TACE | 86 | ≤60 73  >60 13 | 75/11 | 72/14 | NA | 72/14 | ≤400 49  >400 37 | 70/16/0 | 84/2 | 73/13 | ≤5 41  >5 45 | 86/0 |
|  | HR | 174 | ≤60 140  >60 34 | 150/24 | 156/18 | NA | 143/31 | ≤400 89  >400 85 | 127/47/0 | 172/2 | 137/37 | ≤5 76  >5 98 | 174/0 |
| [Wang, H. 2018](44\\WANG H2018.pdf) | TACE | 128 | 52.1±7.2 | 116/7 | 66/57 | NA | 69/56 | 357.00±444.12 | 0/123/0 | 121/2 | 0/123 | 3.84±1.27 | 44/79 |
|  | HR | 143 | 54.5±10.2 | 130/18 | 71/77 | NA | 96/52 | 367.45±474.58 | 0/148/0 | 146/2 | 0/148 | 3.83±1.09 | 84/64 |
| [Wang, Z. 2018](44\\WANG Z2018.pdf) | TACE | 140 | 54.2±9.7 | 121/19 | 140/0 | 0/140 | 72/68 | ≤20 53  >20 87 | NA | NA | 102/38 | ≤5 56  >5 84 | 78/62 |
|  | HR | 140 | 52.6±10.3 | 109/31 | 140/0 | 0/140 | 66/74 | ≤20 51  >20 89 | NA | NA | 109/31 | ≤5 61  >5 79 | 87/53 |
| [Wei, W. 2018](44\\WEI W2018.pdf) | TACE | 116 | 44.0(18.0-75.0) | 106/10 | 94/22 | NA | 50/66 | <25 37  ≥25 79 | 116/0/0 | 116/0 | 116/0 | 5-10 82  >10 34 | 116/0 |
|  | HR | 118 | 48.0(18.0-74.0) | 106/12 | 101/17 | NA | 42/76 | <25 36  ≥25 82 | 118/0/0 | 116/2 | 118/0 | 5-10 97  >10 21 | 118/0 |
| [Liu, S. 2019](44\\LIU S2019.pdf) | TACE | 222 | ≤50 129  >50 93 | 193/29 | 188/34 | NA | NA | ≤20 73  >20 149 | NA | 211/11 | 189/33 | 5.3 | 222/0 |
|  | HR | 222 | ≤50 130  >50 92 | 193/29 | 189/33 | NA | NA | ≤20 75  >20 147 | NA | 210/12 | 182/40 | 5.5 | 222/0 |
| [Qi, Y.-P. 2019](44\\Qi2019.pdf) | TACE | 91 | ≤50 52  >50 39 | 78/13 | 77/14 | NA | 79/12 | ≤400 47  >400 44 | 54/37/0 | NA | 68/23 | ≤5 20  >5 71 | 91/0 |
|  | HR | 109 | ≤50 67  >50 42 | 93/16 | 96/13 | NA | 89/20 | ≤400 59  >400 50 | 76/33/0 | NA | 84/25 | ≤5 34  >5 75 | 109/0 |
| [Wang, Y.-Y. 2019](44\\WANG YY2019.pdf) | TACE | 57 | 55.0±11.0 | 47/10 | 47/10 | 2/55 | 49/8 | <200 32  ≥200 25 | 46/11/0 | 54/3 | 46/11 | 6.00 (2.00-14.00) | 57/0 |
|  | HR | 57 | 56.0±10.0 | 51/6 | 47/10 | 6/51 | 46/9 | <200 34  ≥200 23 | 47/10/0 | 54/3 | 46/11 | 6 .00(2.00-18.00) | 57/0 |
| [Yi, P. S. 2019](44\\YI PS2019.pdf) | TACE | 50 | ≤60 35  >60 15 | 43/7 | 43/7 | NA | 36/14 | ≤400 23  >400 37 | 50/0/0 | NA | 26/24 | ≤5 16  >5 34 | 39/21 |
|  | HR | 52 | ≤60 32  >60 20 | 47/5 | 47/5 | NA | 36/16 | ≤400 34  >400 18 | 52/0/0 | NA | 26/26 | ≤5 14  >5 38 | 28/24 |
| [Zhang, X. P. 2019](44\\Zhang 2019.pdf) | TACE | 185 | 52.00 (47.0–59.0) | 162/23 | 161/24 | NA | 52/133 | ≤400 94  >400 91 | NA | 182/3 | 144/41 | NA | NA |
|  | HR | 134 | 53.00 (47.0–64.0) | 117/17 | 119/15 | NA | 49/85 | ≤400 63  >400 71 | NA | 120/14 | 83/51 | NA | NA |
| [Liu, F.C 2020](44\\LIU FC2020.pdf) | TACE | 90 | 49.9±10.6 | 86/4 | 78/12 | NA | 50/40 | ≤400 57  >400 33 | 0/0/90 | 68/22 | 59/31 | ≤5 21  5-10 44  >5 25 | 74/12 |
|  | HR | 156 | 47.9±10.3 | 140/16 | 144/12 | NA | 103/53 | ≤400 110  >400 46 | 0/0/156 | 141/15 | 101/55 | ≤5 22  5-10 67  >5 67 | 142/14 |
| [Wang, L. 2020](44\\WANG L2020.pdf) | TACE | 199 | ＜50 81  ≥50 118 | 176/23 | 179/20 | NA | 143/56 | ≤400 111  >400 88 | 146/53/0 | 187/12 | 136/63 | ≤5 85  >5 114 | 199/0 |
|  | HR | 199 | ＜50 84  ≥50 115 | 173/26 | 177/22 | NA | 144/55 | ≤400 110  >400 89 | 142/57/0 | 187/12 | 138/61 | ≤5 83  >5 116 | 199/O |
| [Wang, L. 2020](44\\WANG L2021.pdf) | TACE | 123 | ≤60 98  >60 25 | 108/15 | 113/10 | NA | 85/38 | ≤400 81  >400 42 | 0/123/0 | NA | 0/123 | <5 48  ≥5 75 | 55/68 |
|  | HR | 123 | ≤60 87  >60 36 | 111/12 | 112/11 | NA | 85/38 | ≤400 77  >400 46 | 0/123/0 | NA | 0/123 | <5 43  ≥5 80 | 59/64 |
| [Wang, H. 2021](44\\WANG H2021.pdf) | TACE | 69 | 51.1±11.7 | 59/10 | 58/11 | NA | 27/42 | 444.70 (1.50-1210.00) | NA | 69/0 | 41/28 | 13.48±2.99 | 69/0 |
|  | HR | 69 | 50.4±9.8 | 60/9 | 60/9 | NA | 24/45 | 843.20 (1.20-1210.00) | NA | 69/0 | 40/29 | 13.18±2.93 | 69/0 |
| [Qiu, Y.W 2022](44\\QIU2022.pdf) | TACE | 164 | 51.0±12.0 | 138/26 | 135/29 | NA | 86/78 | ≤400 80  >400 84 | NA | 164/0 | 121/43 | 4.70±2.90 | 49/115  (MPVI) |
|  | HR | 164 | 52.0±12.0 | 145/19 | 136/28 | NA | 78/86 | ≤400 62  >400 102 | NA | 164/0 | 112/52 | 5.00±2.90 | 55/109  (MPVI) |
| [Tang, Y.P 2022](44\\TANG YP2022.pdf) | TACE | 152 | ＜50 87  ≥50 65 | 130/22 | 152/0 | 0/152 | 106/46 | ≤400 77  >400 95 | NA | NA | 112/40 | <5- 28  ≥5- 124 | 152/0 |
|  | HR | 319 | ＜50 170  ≥50 149 | 291/28 | 319/0 | 0/319 | 244/75 | ≤400 145  >400 174 | NA | NA | 235/84 | <5- 84  ≥5- 235 | 219/0 |
| [Wang, J. H. 2022](44\\Wang2022.pdf) | TACE | 126 | ＜50 66  ≥50 60 | 113/13 | 114/12 | NA | NA | ≤400 73  >400 53 | 57/69 | NA | 104/22 | <5- 65  ≥5- 61 | NA |
|  | HR | 105 | ＜50 59  ≥50 46 | 97/8 | 100/5 | NA | NA | ≤400 55  >400 50 | 20/85 | NA | 52/53 | <5- 37  ≥5- 68 | NA |
| [Xu, J. X. 2022](44\\Xu2022.pdf) | TACE | 351 | ≤45 116  >45 235 | 306/45 | 114/237 | NA | NA | ≤400 172  >400 179 | NA | NA | 225/126 | NA | 242/109 |
|  | HR | 365 | ≤45 147  >45 218 | 318/47 | 120/245 | NA | NA | ≤400 158  >400 207 | NA | NA | 199/166 | NA | 290/75 |
| Xiang,C 2024 | TACE | 137 | 52 （19–80） | 123/14 | 116/21 | NA | 90/47 | ≤400 73  >400 64 | NA | NA | 102/35 | NA | NA |
|  | HR | 109 | 51 （18–79） | 94/15 | 93/16 | NA | 76/33 | ≤400 57  >400 52 | NA | NA | 82/27 | NA | NA |
| Ma.Z 2024 | TACE | 42 | 61.69±9.27 | 31/11 | 30/12 | NA | NA | 143.5  (70.25–315.5) | NA | 11/31 | NA | 7.15±3.05 | 42/0 |
|  | HR | 51 | 62.78±9.97 | 37/14 | 38/13 | NA | NA | 192  (71–425.5) | NA | 33/18 | NA | 6.662±3.61 | 51/0 |
| [Wang, L. 2017](44\\WANG LM2017.pdf) | TACE | 42 | 51.38 ± 10.89 | 8/34 | NA | NA | 4/38 | 25/17 | NA | 42/0 | 38/4 | 6.15 ± 3.65 | 42/0 |
|  | RT | 44 | 51.32 ± 11.21 | 5/39 | NA | NA | 4/40 | 34/10 | NA | 44/0 | 41/3 | 5.63 ± 2.73 | 44/0 |
|  | HR | 50 | 57.22 ± 11.14 | 5/45 | NA | NA | 2/48 | 32/18 | NA | 50/0 | 46/4 | 5.71 ± 2.60 | 50/0 |
| [Wang, L. 2019](44\\Wang2019.pdf) | TACE | 71 | 51.59±10.84 | 60/11 | 58/13 | NA | NA | 15/30/26 | NA | NA | 63/8 | 6.57±3.65 | 71/0 |
|  | RT | 46 | 50.98±10.53 | 43/3 | 38/8 | NA | NA | 18/18/10 | NA | NA | 15432 | 5.39±2.74 | 46/0 |
| [Shen, P. C. 2020](44\\SHEN PC2020.pdf) | TACE | 52 | ≤50 20  >50 32 | 37/15 | 44/8 | NA | 42/10 | ≤400 29  >400 23 | NA | 48/8 | NA | ≤5 27  >5 25 | 52/0 |
|  | Sorafenib | 36 | ≤50 11  >50 25 | 28/8 | 27/9 | NA | 26/10 | ≤400 17  >400 19 | NA | 34/2 | NA | ≤5 21  >5 15 | 36/0 |
|  | HR | 49 | ≤50 22  >50 27 | 36/13 | 41/8 | NA | 33/16 | ≤400 25  >400 24 | NA | 30/6 | 13/29 | ≤5 30  >5 19 | 49/0 |
| [Lin, K. 2022](44\\Lin2-022.pdf) | TACE | 142 | 53.1 (11.7) | 125/17 | 134/8 | NA | 101/41 | 77/65 | NA | NA | 119/23 | 8.49 (3.68) | NA |
|  | TACE+TKI | 57 | 48.6 (12.7) | 47/10 | 47/10 | NA | 38/19 | 29/28 | NA | NA | 47/10 | 8.85 (3.94) | NA |

TACE, transhepatic arterial chemoembolization; HAIC, hepatic arterial infusion chemotherapy; RT, radiotherapy; S, single; M, multiple; BCLC, Barcelona Clinic Liver Cancer; TNM stage: American Joint Committee on Cancer TNM staging system, 7th edition; pTNM: pathological TNM; NA, not available.

Supplementary Table 4 Specific quality assessment information for the cohort studies

| cohort | Selection | | | | Comparability | Outcome | | | Score |
| --- | --- | --- | --- | --- | --- | --- | --- | --- | --- |
|  | 1 | 2 | 3 | 4 | 5 | 6 | 7 | 8 |  |
| [Hsiao, J.-H. 2017](44\\HSIAO2017.pdf) | * | * | * | * | * | * | * |  | 7 |
| [Hamada, T. 2020](44\\HAMADA2020.pdf) | * | * | * | * | * | * | * |  | 7 |
| [Feng,M 2017](44\\LI S2020.pdf) | * | * | * | * | ** | * | * |  | 8 |
| [Wang, L. 2020](44\\WANG L2020 (2).pdf) | * | * | * | * | * | * | * |  | 7 |
| [Zhang, W. 2014](44\\zhang w 2014.pdf) | * | * |  | * | ** | * | * |  | 7 |
| [Xia, F. 2016](44\\XIA F 2016.pdf) | * | * |  | * | * | * | * |  | 6 |
| [Li，Jiang 2016](44\\Li Jiang2016.pdf) | * | * |  | * | ** | * | * |  | 7 |
| [Liao, Y.D 2017](44\\LIAO YD2017.pdf) | * | * |  | * | * | * | * |  | 6 |
| [Huang, Y. 2019](44\\HUANG Y2019.pdf) | * | * |  | * | ** | * | * |  | 7 |
| [Zhang, X.-P. 2019](44\\ZHANG XP2019.pdf) | * | * |  | * | * | * | * |  | 6 |
| [Li, Q. L 2021](44\\LI QL2021.pdf) | * | * |  | * | ** | * | * |  | 7 |
| [Bai, S. 2022](44\\Bai 2022.pdf) | * | * | * | * | ** | * | * |  | 8 |
| Dai,M 2023 | * | * | * | * | ** | * | * |  | 8 |
| [Wang,TH,  2008](44\\WANG TH2008.pdf) | * | * | * | * | * | * | * |  | 8 |
| [Li, F. 2014](44\\LI F2015.pdf) | * | * | * | * | * | * | * |  | 7 |
| [Dong, Z. R. 2015](44\\Dong 2015.pdf) | * | * | * | * | * | * | * |  | 7 |
| [Sun, J. J. 2016](44\\SUN JJ2015.pdf) | * | * | * | * | * | * | * |  | 7 |
| [Li, C. 2017](44\\LI C2017.pdf) | * | * | * | * | ** | * | * |  | 8 |
| [Ye, J. Z. 2017](44\\Ye2017.pdf) | * | * | * | * | * | * | * |  | 7 |
| [Wang, H. 2018](44\\WANG H2018.pdf) | * | * | * | * | ** | * | ***** |  | 8 |
| [Liu, S. 2019](44\\LIU S2019.pdf) | * | * | * | * | ** | * | * |  | 8 |
| [Qi, Y.-P. 2019](44\\Qi2019.pdf) | * | * | * | * | * | * | * |  | 7 |
| [Wang, Y.-Y. 2019](44\\WANG YY2019.pdf) | * | * | * | * | ** | * | * |  | 8 |
| [Yi, P. S. 2019](44\\YI PS2019.pdf) | * | * | * | * | * | * | * |  | 7 |
| [Zhang, X. P. 2019](44\\Zhang 2019.pdf) | * | * |  | * | * | * | * |  | 6 |
| [Liu, F.C 2020](44\\LIU FC2020.pdf) | * | * | * | * | * | * | * |  | 7 |
| [Wang, L. 2020](44\\WANG L2020.pdf) | * | * | * | * | ** | * | * |  | 8 |
| [Wang, L. 2020](44\\WANG L2021.pdf) | * | * | * | * | * | * | * |  | 7 |
| [Wang, H. 2021](44\\WANG H2021.pdf) | * | * | * | * | ** | * | * |  | 8 |
| [Qiu, Y.W 2022](44\\QIU2022.pdf) | * | * | * | * | * | * | * |  | 7 |
| [Tang, Y.P 2022](44\\TANG YP2022.pdf) | * | * |  | * | * | * | * |  | 6 |
| [Wang, J. H. 2022](44\\Wang2022.pdf) | * | * | * | * | * | * | * |  | 7 |
| [Xu, J. X. 2022](44\\Xu2022.pdf) | * | * | * | * | ** | * | * |  | 8 |
| Xiang,C 2024 | * | * | * | * | ** | * | * |  | 8 |
| Ma.Z 2024 | * | * | * | * | ** | * | * |  | 8 |
| [Wang, L. 2017](44\\WANG LM2017.pdf) | * | * | * | * | * | * | * |  | 7 |
| [Wang, L. 2019](44\\Wang2019.pdf) | * | * | * | * | ** | * | * |  | 8 |
| [Shen, P. C. 2020](44\\SHEN PC2020.pdf) | * | * | * | * | * | * | * |  | 7 |
| [Lin, K. 2022](44\\Lin2-022.pdf) | * | * | * | * | * | * | * |  | 7 |
| Wang, SN  2014 | * | * |  | * | ** | * | * |  | 7 |
| Qi, YP  2019 | * | * | * | * | * | * | * |  | 7 |

1. Representativeness of the exposed cohort; 2-Selection of the non-exposed cohort; 3-Exposure ascertainment; 4- Demonstration that outcome of interest was not present at start of study; 5-Comparability of cohorts on the basis of design or analysis; 6-Outcome assessment; 7-Was follow-up long enough for outcomes to occur? 8-Adequacy of cohort follow-up.

Supplementary Figure S1


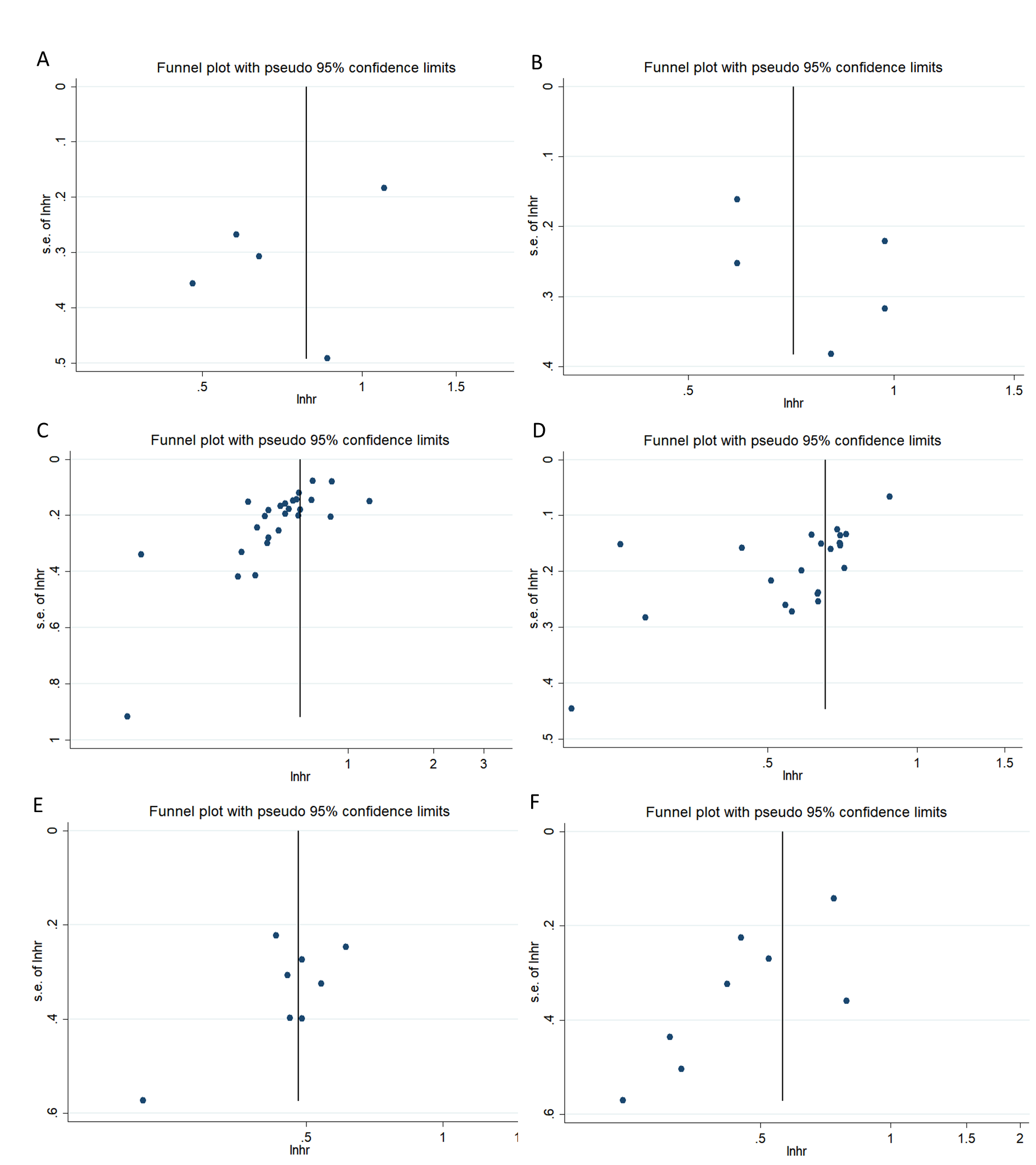


Funnel plots for the assessment of publication bias HAIC OS (A) DFS (B), TACE OS (C) DFSD(D), TKIs OS (E) DFS (F).
